# Poly(A) binding KPAF4/5 complex stabilizes kinetoplast mRNAs in *Trypanosoma brucei*

**DOI:** 10.1093/nar/gkaa575

**Published:** 2020-07-02

**Authors:** Inna Aphasizheva, Tian Yu, Takuma Suematsu, Qiushi Liu, Mikhail V Mesitov, Clinton Yu, Lan Huang, Liye Zhang, Ruslan Aphasizhev

**Affiliations:** Department of Molecular and Cell Biology, Boston University Medical Campus, Boston, MA 02118, USA; Department of Molecular and Cell Biology, Boston University Medical Campus, Boston, MA 02118, USA; Department of Molecular and Cell Biology, Boston University Medical Campus, Boston, MA 02118, USA; Department of Molecular and Cell Biology, Boston University Medical Campus, Boston, MA 02118, USA; Department of Molecular and Cell Biology, Boston University Medical Campus, Boston, MA 02118, USA; Department of Physiology and Biophysics, School of Medicine, University of California, Irvine, CA 92697, USA; Department of Physiology and Biophysics, School of Medicine, University of California, Irvine, CA 92697, USA; School of Life Science and Technology, ShanghaiTech University, Shanghai 201210, China; Department of Molecular and Cell Biology, Boston University Medical Campus, Boston, MA 02118, USA; Department of Biochemistry, Boston University Medical Campus, Boston, MA 02118, USA

## Abstract

In *Trypanosoma brucei*, mitochondrial pre-mRNAs undergo 3′-5′ exonucleolytic processing, 3′ adenylation and uridylation, 5′ pyrophosphate removal, and, often, U-insertion/deletion editing. The 3′ modifications are modulated by pentatricopeptide repeat (PPR) Kinetoplast Polyadenylation Factors (KPAFs). We have shown that KPAF3 binding to the 3′ region stabilizes properly trimmed transcripts and stimulates their A-tailing by KPAP1 poly(A) polymerase. Conversely, poly(A) binding KPAF4 shields the nascent A-tail from uridylation and decay thereby protecting pre-mRNA upon KPAF3 displacement by editing. While editing concludes in the 5′ region, KPAF1/2 dimer induces A/U-tailing to activate translation. Remarkably, 5′ end recognition and pyrophosphate hydrolysis by the PPsome complex also contribute to mRNA stabilization. Here, we demonstrate that KPAF4 functions as a heterodimer with KPAF5, a protein lacking discernable motifs. We show that KPAF5 stabilizes KPAF4 to enable poly(A) tail recognition, which likely leads to mRNA stabilization during the editing process and impedes spontaneous translational activation of partially-edited transcripts. Thus, KPAF4/5 represents a poly(A) binding element of the mitochondrial polyadenylation complex. We present evidence that RNA editing substrate binding complex bridges the 5′ end-bound PPsome and 3′ end-bound polyadenylation complexes. This interaction may enable mRNA circularization, an apparently critical element of mitochondrial mRNA stability and quality control.

## INTRODUCTION

Hemoflagellate protists *Trypanosoma brucei* (*T. brucei* sp.) cause human sleeping sickness and animal trypanosomiasis (Nagana), which endanger public health and economy in sub-Saharan Africa. A single mitochondrion of these parasites contains a bipartite genome composed of catenated 23-kb maxicircles and 1-kb minicircles. A few maxicircles encode 9S and 12S ribosomal RNAs (rRNAs), six protein-coding and 12 pseudogenes, a *trans*-acting MURF2-II, and *cis*-acting CO_2_ guide RNAs (gRNA). Approximately 5000 minicircles produce gRNAs that direct U-insertion/deletion editing of pseudogene transcripts (1–4). The 5′ termini of messenger, ribosomal, and guide RNA precursors are set by transcription initiation, but only mature gRNAs maintain nucleoside triphosphate incorporated at the start site. In contrast, mRNAs and rRNA 5′ ends are modified by the PPsome (Table 1). This complex of MERS1 pyrophosphohydrolase and MERS2 pentatricopeptide repeat (35-amino acids, PPR) RNA binding factor removes pyrophosphate and stabilizes mature molecules by an unknown mechanism (5). Maxicircle- and minicircle-encoded 3′-extended precursors are processed by antisense RNA-controlled 3′-5′ exonucleolytic trimming, which is followed by adenylation of pre-mRNAs, or rRNA and gRNA uridylation (6). Trimming is accomplished by the mitochondrial 3′ processome (MPsome), a stable complex of KDSS1 3′-5′ exonuclease (7), KRET1 TUTase (8), and several subunits of undefined functions (9). The non-templated 3′ mRNA modifications are temporally separated and distinct in structure and function: KPAP1 poly(A) polymerase adds a short (15–30 nt) A-tail prior to, or concurrent with, initial editing events in the 3′ region. The short A-tail is dispensable for pre-edited, but is required for edited mRNA stability (10,11). Conversely, post-editing extension of the A-tail into a long (100–300 nt) A/U-heteropolymer marks fully-edited mRNA for translation (12). The coupling between mRNA editing status, the timing of 3′ extensions, and their distinct roles suggest the existence of a surveillance mechanism that potentially: (i) stimulates addition and enables A-tail's stabilizing function; (ii) monitors editing initiation and completion as sequence changes proceed from the 3′ to the 5′ region and (iii) induces 3′ A/U-tailing upon editing cessation in the 5′ region. These tasks have been attributed to PPR Kinetoplast Polyadenylation Factors KPAF3 (6), KPAF4 (13) and KPAF1/2 (12), respectively. Discovered in land plants (14), the helix-turn-helix PPR motif recognizes a single nucleoside via side chains occupying cardinal positions 5 and 35 of the repeat (or the last position in a longer repeat). An array of adjacent PPR motifs may recognize a specific RNA sequence to modulate various modification and degradation enzymes (15–17). In this context, KPAF3 binding to G-rich pre-edited mRNAs is thought to stabilize these species prior to adenylation (6), and to stimulate A-tailing by KPAP1 poly(A) polymerase (6,10). It has been proposed that KPAF3 displacement by editing events licenses mRNA stabilization to the short A-tail bound by the poly(A) binding PPR factor KPAF4 (6). Finally, a signaling event that senses editing completion in the 5′ region and triggers short A-tail extension into an A/U-tail by KPAP1 poly(A) polymerase, KRET1 TUTase and KPAF1/2 heterodimer has been envisaged (12). Here, we demonstrate that KPAF4 interacts directly with KPAF5 (Tb927.3.2670), a novel 29 kDa protein lacking known motifs or sequence similarities beyond the class of Kinetoplastea. KPAF5, originally detected by co-purification with KPAF4 (13), is essential for the viability of insect (procyclic, PF) and mammalian (bloodstream, BF) parasite forms, and for KPAF4 and KPAP1 maintenance in the cell. We present evidence that KPAF4/5 poly(A) binding complex stabilizes never-edited, pre-edited, and edited mRNAs, but not rRNAs or gRNAs. Furthermore, our data suggest that RNA editing substrate binding complex (RESC) bridges the 5′ end-bound PPsome and 3′ end-bound polyadenylation (KPAC) complexes. This interaction likely enables mRNA circularization during the editing process, thereby stabilizing mRNA prior to A/U-tailing. Fittingly, we demonstrate that knockdowns of proximal RESC subunits 10 and 13 (TbRGG2) (18) negatively impact the steady-state level of a never-edited transcript. These findings associate RESC with a general mRNA stabilization-by-circularization mechanism.

**Table 1.** Abbreviations and definitions

Name	Definition	Function
KPAC	Kinetoplast polyadenylation complex	mRNA 3′ stabilization and adenylation
PPsome	Pyrophosphohydrolase complex	mRNA 5′ end modification and stabilization
MPsome	Mitochondrial processome	3′-5′ processing and decay
RECC	RNA editing catalytic complex	mRNA editing reactions
RESC	RNA editing substrate binding complex	mRNA editing and gRNA stabilization
RESC1/2	Guide RNA binding RESC module	Guide RNA stabilization
REH2C	RNA editing helicase 2 complex	Editosome remodeling, gRNA binding
PPR	pentatricopeptide (35 aa) repeat	Helix-turn-helix RNA binding motif
KPAP1	Kinetoplast poly(A) polymerase	mRNA adenylation
KPAFs	Kinetoplast polyadenylation factors	mRNA 3′ end modification and stabilization
KDSS1	RNase II/RNB-type 3′-5′ exonuclease	MPsome subunit; RNA degradation
KRET1	Terminal uridylyl transferase (TUTase)	MPsome subunit; U-tailing
MERS1	NUDIX pyrophosphohydrolase	PPsome catalytic subunit, 5′ PPi removal
MERS2	PPR protein	PPsome RNA binding subunit
Pan-edited	A transcript undergoing massive editing	Translatable reading frame
Partially-edited	Editing intermediates	Interrupted reading frame, untranslatable
Moderately-edited	A transcript with a few editing sites confined to a limited region.	Translatable reading frame
Never-edited	Contains encoded open reading frame, does not require editing.	Translatable reading frame

## MATERIALS AND METHODS

### RNA interference and protein expression

Plasmids for RNAi knockdowns were generated by cloning an ∼500-bp gene fragment into p2T7–177 vector for tetracycline-inducible expression of double-stranded RNA (19). Linearized constructs were transfected into a procyclic 29–13 or into bloodstream ‘single marker’ transgenic derivatives of the Lister 427 *T. brucei* strain (20). For inducible protein expression, full-length genes were cloned into pLew-MHTAP vector and transfected into 29–13 PF (21). For BioID experiments in PF, full-length genes were cloned into the same vector with the C-terminal TAP tag replaced by a mutated BirA* ligase from *Escherichia coli* (22). DNA oligonucleotides are listed in Supplementary Data.

### Purification of recombinant KPAF4/5, KPAF5 and antibody production

KPAF4 and KPAF5 with truncated predicted mitochondrial importation signal peptides (positions −25 and −60, respectively) were co-expressed in pETDuet-1 vector and purified from *E. coli* BL21 (DE3) STAR by sequential metal affinity, Strep-Tactin and size exclusion chromatographic steps. Full-length KPAF5 was expressed in *E. coli* BL21 (DE3) STAR as a C-terminal fusion with 6-His tag. The protein was purified to apparent homogeneity by metal affinity and cation exchange chromatography. Rabbit polyclonal antibody was raised against the recombinant protein and purified with immobilized antigen. Details are provided in Supplementary Data.

### Biochemical analysis

Mitochondrial isolation, glycerol gradient fractionation, native gel, total RNA isolation, northern and western blotting, qRT-PCR, and tandem affinity purification were performed as described (23). The change in relative abundance was calculated from qRT-PCR, northern or western blotting data as a ratio between RNA or protein of interest and normalization control in mock-induced cells. For BioID, biotinylated proteins were purified from mitochondrial fraction (13).

### Coupled *in vitro* transcription-translation in reticulocyte lysate

KPAF4 and KPAF5 were co-synthesized using 100 ng of plasmid and 5 μCi of [^35^S] methionine in a 50 μl reaction with the TNT system (Promega). Co-precipitation was performed with Dynabeads Protein G (Thermo Fisher) conjugated with KPAF5 polyclonal antibody.

### Protein identification by LC−MS/MS

Affinity-purified complexes were sequentially digested with LysC peptidase and trypsin. LC-MS/MS was carried out by nanoflow reversed phase liquid chromatography (RPLC) using an UltiMate 3000 RSLC (Thermo Scientific) coupled on-line to an Orbitrap Fusion Lumos mass spectrometer (Thermo Scientific). A cycle of full FT scan mass spectrum (*m*/*z* 375–1500, resolution of 60 000 at *m*/*z* 400) was followed by MS/MS spectra acquired in the linear ion trap for 3 s at top speed with normalized collision energy (HCD, 30%). Following data extraction to an MGF format using MSConvert (ProteoWizard), the resultant peak lists for each LC−MS/MS experiment were submitted to Protein Prospector (UCSF) for database searching (24). Each project was searched against a normal form concatenated with the random form of the *T. brucei* database (http://tritrypdb.org/tritrypdb/). The mass accuracies for parent ions and fragment ions were set as ±10 ppm and 0.6 Da, respectively. Trypsin was set as the enzyme, with a maximum of two missed cleavages allowed. Cysteine carbamidomethylation was set as a fixed modification, and protein N-terminal acetylation, methionine oxidation, and N-terminal conversion of glutamine to pyroglutamic acid were selected as variable modifications.

### Messenger RNA 3′ extensions sequencing (Tail-Seq), crosslinking-affinity purification-sequencing (eCLAP-Seq) and global mitochondrial RNA-Seq

For Tail-Seq, 5 μg of total cellular RNA was circularized with 30U of T4 RNA ligase 1 in 50 μl at 14°C for 16 h and subsequently digested with 5 U of RNase R (Epicenter) for 10 min at 37°C to remove linear RNAs. Flanking termini and non-encoded extensions were amplified with gene-specific primers. Three replicate libraries were sequenced on Illumina platform in 150 bp paired-end mode (25). For eCLAP, parasites growing in SDM-79 media were transferred into a VARI-X-LINK irradiation chamber and irradiated at 254 nm for 20 s at maximum intensity. Affinity purification of RNA−protein adducts and RNA-Seq library preparation have been performed as described (23), with modifications outlined in Supplementary Data. For global RNA-Seq, the random-primed cDNA library was generated with total RNA extracted from Renografin density gradient-enriched PF mitochondrial fraction (23). The RNA-Seq library has been generated with a NEBNext^®^ Ultra™ RNA Library Prep Kit.

### Tail-Seq and eCLAP-Seq data analysis pipelines

For Tail-Seq, the 5′ and 3′ encoded regions flanking non-templated 3′ additions were removed and mRNA identity assigned with default parameters in Cutadapt (v2.5) (26). Nucleotide frequencies for each read were calculated through an in-house Perl script; tails with A+T content lower than 90% were discarded. Positional nucleotide frequency and tail length distribution were calculated with an in-house Perl script. Graphs were created by setting the encoded 3′ end as zero and plotting the relative nucleotide position on the X-axis, and the corresponding nucleotide frequency and length distribution on the Y-axis. For eCLAP, FASTQ files were decompressed and subjected to FastQC (v0.11.9) quality check and adapter identification (27). Adapters were trimmed with Cutadapt, and processed reads longer than 25 nt were retained. The 10 nt sequencing barcodes were removed with a FASTX-Toolkit (hannonlab.cshl.edu/fastx_toolkit/). Adapter-trimmed read pairs were merged into a single read via PEAR (0.9.10) (28) with the minimum assembly length of 15 nt, and filtered against *T. brucei* 427 nuclear genome (www.tritrypdb.org). The resultant datasets were mapped to maxicircle DNA (Genbank ID: M94286.1) and to edited mRNA sequences (29). The read mapping was performed using Bowtie2 (30) and BWA (v0.7.11) (31) with default parameters. The output SAM files from the two aligners were merged by Samtools (v1.10) (32). The total read depth for each nucleotide position was calculated with an in-house Perl script. A partially-mapped read was included if: (i) it contains a mapped part followed by an unmapped part; (ii) the mapped part aligns to mRNA without mismatches or indels; (iii) the mapping position lies within 50 nt from the 5′ or 10 nt from the 3′ terminus and (iv) the unmapped part contains a string of the same nucleotides. The positional read count and masked nucleotide count were visualized by plotting the mRNA coordinates on the X-axis and the corresponding read count on the Y-axis. Positional and masked nucleotide read counts from individual mRNAs were aggregated to show characteristics typical of a group: i.e., never-edited, pan-edited, or moderately-edited. To eliminate bias introduced by individual transcript's steady-state abundance, global mitochondrial RNA-Seq was performed and an mRNA normalization factor was calculated based on 12S rRNA. The expression level was calculated as ‘transcript per million (TPM),’ defined by the fraction of one million reads that mapped to 1000 nt of the transcript. The positional read count and masked nucleotide count in each eCLAP dataset were normalized by dividing the raw read count with the normalization factor. The normalized positional read count and masked nucleotide count were aggregated for never-edited mRNAs, moderately-edited mRNAs, and pan-edited mRNAs.

### Interaction networks

The relative target-to-bait abundance was calculated by dividing the total peptide count by protein molecular mass. To visualize interactions, the nodes were defined by either individual proteins or groups of proteins, and the edge length between nodes was determined based on the Fruchterman−Reingold (FR) layout algorithm using an in-house R script with the igraph package (v1.2.4.2, https://igraph.org/). The relative abundance for the node pairs was normalized by the arithmetic mean, assuming the mean of the normalized values as 1. The normalized abundance value served as multiplier in the FR algorithm, where values greater than 1 increase attraction between nodes, and values <1 decrease attraction.

## RESULTS

### KPAF4/5 interacts with polyadenylation and editing complexes

**Figure 1.**
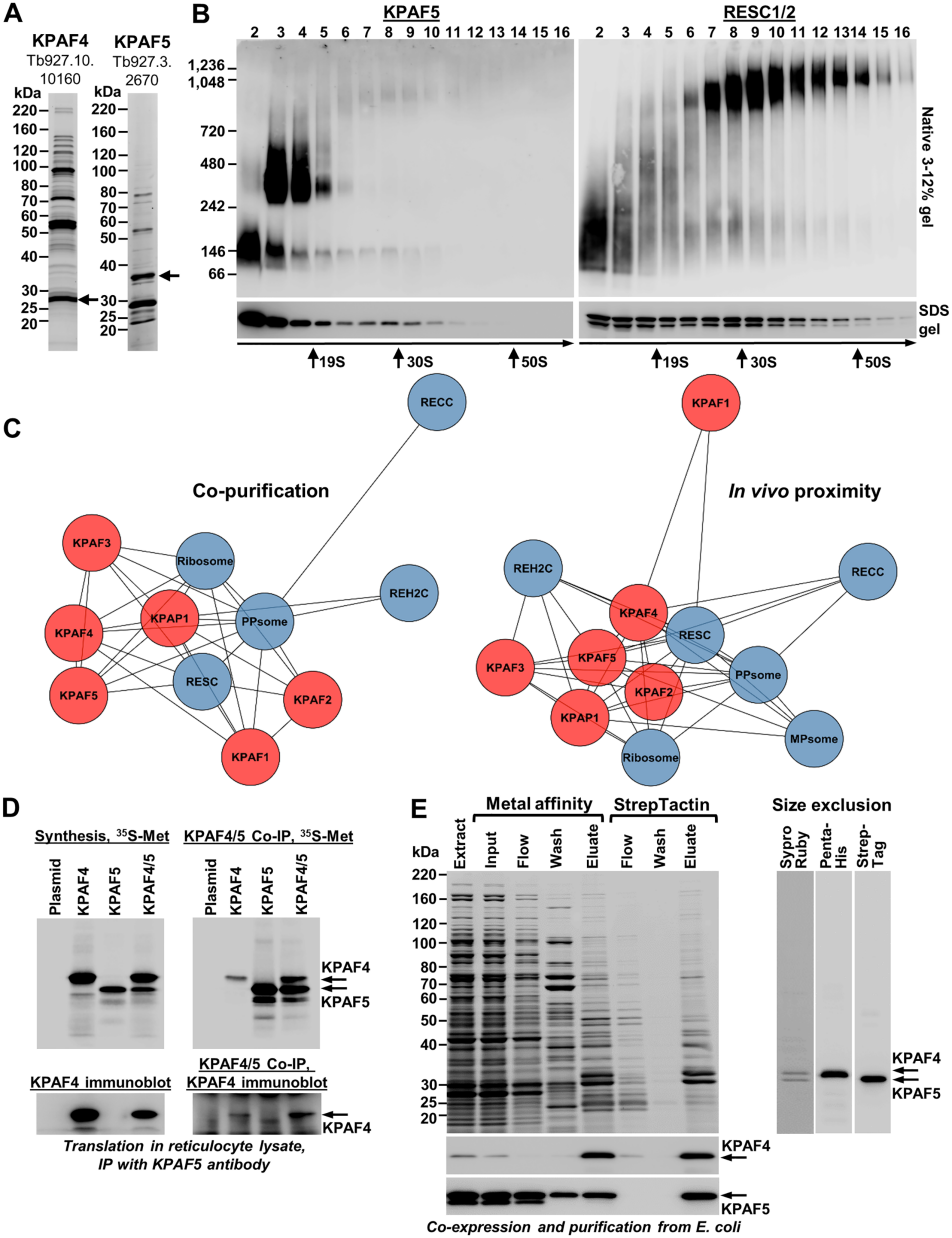
Kinetoplast polyadenylation factors 4 and 5 form a stable heterodimer involved in higher-order interactions. (**A**) Tandem affinity purification of KPAF4 and KPAF5. Final fractions were separated on 8–16% SDS gel and stained with Sypro Ruby. Bait proteins retaining calmodulin binding peptide and 6-His tags after release by TEV protease (21) are indicated by arrows. (**B**) Mitochondrial extract was separated for 5 h at 178 000 g in a 10–30% glycerol gradient. Each fraction was resolved on 3–12% Bis–Tris native gel (upper panels) and 8–16% SDS gel (lower panels). Positions of native protein standards are indicated. KPAF5 and RESC1/2 were visualized by immunoblotting with polyclonal antibodies. Thyroglobulin (19S) and bacterial ribosomal subunits were used as apparent S-value standards. (**C**) Affinity co-purification (left) and *in vivo* proximity biotinylation (BioID (22), right) interaction networks involving KPAF4/5. Rapid pulldowns were performed with TAP-tagged KPAF1, KPAF2, KPAF3, KPAF4, KPAF5, KPAP1 and MERS2 proteins. BioID experiments were carried out with BirA*-tagged MERS1, MERS2, KPAP1, KPAF3, KPAF4, KPAF5, RESC2, RESC5, RESC7, RESC3, RESC18 and RESC19 proteins (41). KPAP1 poly(A) polymerase and KPAF polyadenylation factors are shown individually in red background, while RECC, RESC, REH2C, PPsome (4) and the ribosome (45,46) were collapsed into single teal-colored nodes. Shorter distance between the nodes reflects stronger predicted interaction. (**D**) *In vitro* reconstitution of KPAF4/5 heterodimer. Synthesis: individual proteins, or their combination, were synthesized in a coupled transcription–translation reticulocyte system supplemented with [^35^S]methionine. KPAF5 Co-IP: Immunoprecipitations were performed with immobilized anti-KPAF5 polyclonal antibody. Co-precipitated proteins were separated on 8–16% SDS PAGE and exposed to phosphor storage screen, or visualized by immunoblotting with antibodies against KPAF4. (**E**) Co-expression and purification of KPAF4 (C-terminal 6-His tag) and KPAF5 (C-terminal Twin-Strep tag) from bacteria. KPAF4 and KPAF5 were detected by immunoblotting with tag-specific antibodies. Extract: total cell lysate; Input: cleared cell lysate. Flow: unbound material. Eluate from Talon metal affinity column was applied directly to StrepTactin column. Eluate from StrepTactin column was concentrated and applied to Superose 12 size exclusion column. Chromatographic profile and KPAF4/5 apparent molecular mass calculation are shown in Supplementary Figure S1.

Attempts to purify recombinant poly(A) binding factor KPAF4 led to a hypothesis that a binding partner may be required to stabilize this polypeptide consisting almost entirely of seven adjacent PPR repeats (13). To identify potential interacting proteins, we expressed C-terminally TAP-tagged KPAF4 in a procyclic Lister 427 29-13 (TetR T7RNAP) strain of *T. brucei* (20) and analyzed the tandem affinity purified fraction (Figure 1A, left panel) by LC–MS/MS. A predicted mitochondrially-targeted protein Tb927.3.2670, termed Kinetoplast Polyadenylation Factor 5 (KPAF5), has been detected with high coverage (71%) and relative abundance (140% spectral counts versus the bait, Supplementary Table S1). To verify KPAF4/5 co-purification, a reciprocal analysis has been performed with full-length KPAF5 (Figure 1A, right panel, and Supplementary Table S1). To assess the apparent molecular mass of a putative KPAF4/5 complex, we fractionated mitochondrial extract from the parental Lister 427 29-13 strain on 10–30% glycerol gradient and separated individual fractions by 3–12% native gel. Immunoblotting with polyclonal antibodies against recombinant KPAF5 detected two major particles with apparent molecular masses of ∼100 kDa and ∼350 kDa (Figure 1B, left panel), resembling those described for KPAF4 (13). A minor KPAF5 fraction migrating at ∼1 MDa co-fractionated with RNA editing substrate binding complex (RESC), which was visualized with antibodies against RESC1/2 (Figure 1B, right panel).

To investigate KPAF5 interactions with mitochondrial RNA processing complexes, we purified known components of the kinetoplast polyadenylation complex (KPAC), and representative subunits of RNA editing catalytic (RECC), substrate binding (RESC), and KREH2 RNA helicase (REH2C) complexes, which collectively constitute the RNA editing holoenzyme (4). In addition, 5′ end modification (PPsome) and 3′ end trimming (MPsome) complexes and ribosomal subunits were isolated. In an orthogonal approach, the relative proximity of representative components of RNA processing complexes was ascertained by *in vivo* biotinylation (BioID) (22). Co-purification and *in vivo* proximity networks consistently predicted a strong interaction between KPAF4 and KPAF5, and reflected an overall affinity between KPAC, RESC, and PPsome complexes (Figure 1C). Conversely, RECC, REH2C and MPsome proteins were underrepresented, or fell below the peptide count threshold, in KPAC and RESC affinity purifications.

To test whether KPAF4 and KPAF5 form a heterodimer, we co-synthesized C-terminally 6-His tagged KPAF4 and Twin-Strep tagged KPAF5 in the reticulocyte coupled transcription-translation system (Figure 1D). Predicted mitochondrial importation peptides were removed from both proteins. Co-immunoprecipitation with KPAF5 antibody demonstrated formation of a stable protein-protein interaction between KPAF4 and KPAF5. We note that attempted co-synthesis with KPAP1 poly(A) polymerase did not detect a stable interaction with KPAF4/5, or with individual KPAF5, under the experimental conditions used. Co-expression in *E. coli* and sequential metal affinity, streptavidin affinity, and size exclusion chromatographic purifications confirmed that KPAF4 and KPAF5 indeed constitute a stoichiometric complex (Figure 1E). The apparent KPAF4/5 molecular mass inferred from size exclusion chromatography profile stands at ∼90 kDa (Supplementary Figure S1), which closely matches ∼68 kDa calculated mass of the KPAF4/5 heterodimer assuming it is a 1:1 complex. It seems likely that the reconstituted KPAF4/5 resembles the smaller ∼100 kDa complex detected in mitochondrial extract (Figure 1B, fraction 2) while the larger particles (Figure 1B, fractions 3–4 and 7–10) reflect higher-order assemblies involving KPAF4/5, such as polyadenylation and/or RESC complexes (Figure 1B, C).

### KPAF5 is essential for normal growth of procyclic and bloodstream parasite forms

**Figure 2.**
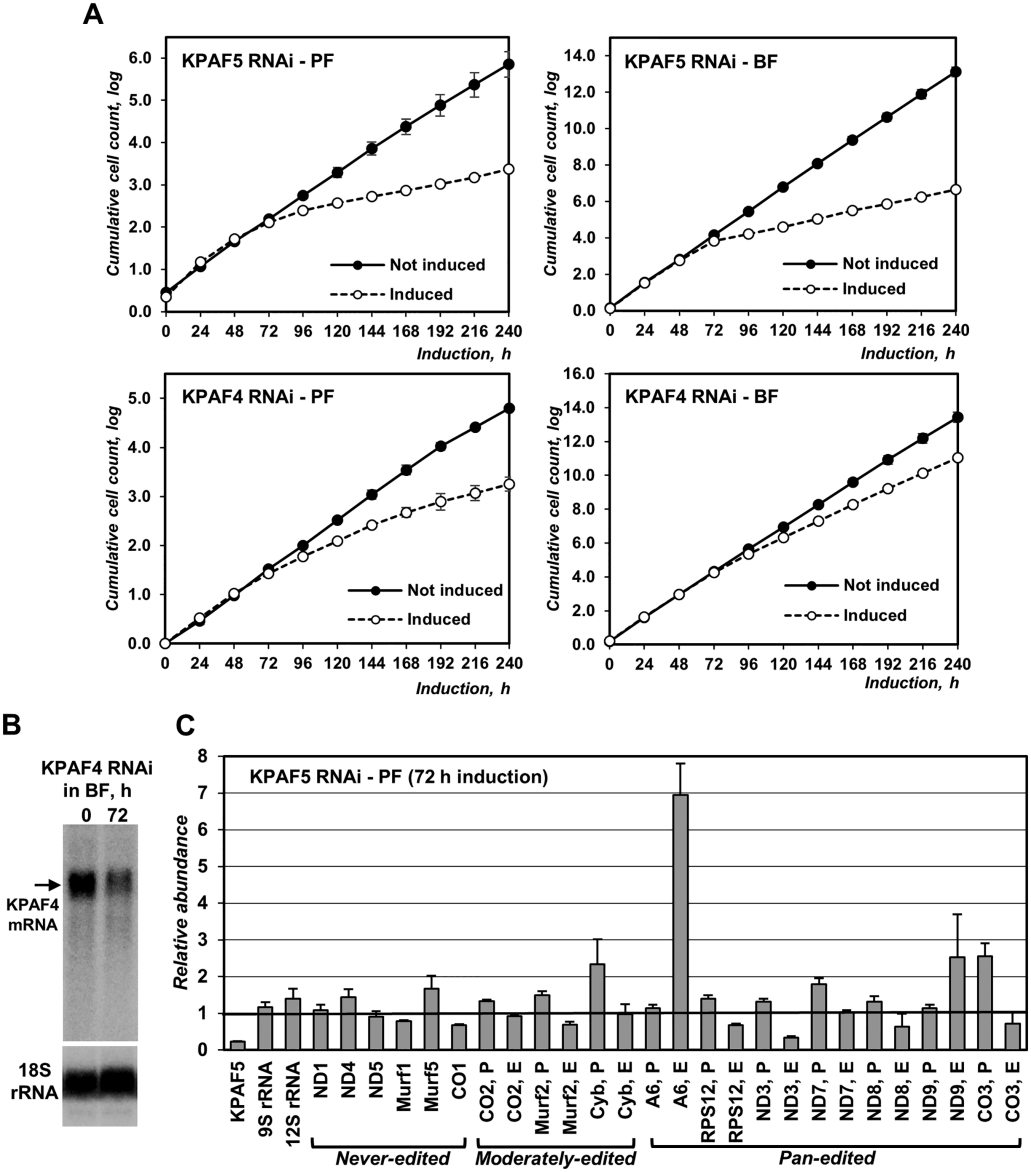
KPAF5 is essential for viability of procyclic and bloodstream parasite forms. (**A**) Growth kinetics of procyclic (PF) and bloodstream (BF) parasite cultures after mock treatment and RNAi induction with tetracycline. Data from three independent experiments are shown as mean ± s.d. (**B**) Northern blotting analysis of KPAF4 mRNA downregulation by inducible RNAi in BF. (**C**) Real-time RT-PCR analysis of RNAi-targeted KPAF5 mRNA, mitochondrial rRNAs and mRNAs. RNA levels were normalized to β-tubulin mRNA. RNAi was induced for 72 h. Error bars show the standard deviation from at least three biological replicates. The thick line at ‘1’ reflects no change in relative abundance; bars above or below represent an increase or decrease, respectively. P, pre-edited mRNA; E, edited mRNA.

The impacts of KPAF4 and KPAF5 RNAi knockdowns on parasite viability were examined in procyclic and bloodstream forms of *T. brucei*. In agreement with the previous study (13), inducible KPAF4 RNAi triggered a moderate cell growth inhibition phenotype in PF, indicating that KPAF4 is essential for normal cellular function. The RNAi effect was less pronounced in BF (Figure 2A) notwithstanding an efficient KPAF4 mRNA (Figure 2B) and protein (Figure 5B) depletion. On the other hand, KPAF5 repression caused a severe growth inhibition in both forms after 72 h of RNAi induction and massive cell death beyond 120 h. These results demonstrate that KPAF5 plays an essential function(s) in insect and mammalian proliferative forms. Quantitative RT-PCR analysis of RNA samples isolated at 72 h after KPAF5 RNAi induction in PF demonstrated divergent transcript-specific consequences at the mRNA level. Downregulation of some pan-edited (RPS12, ND3, ND8, and CO3) mRNAs was accompanied by upregulation of their respective pre-edited forms, which may indicate editing-dependent influence of altered 3′ modifications on mRNA stability. Conversely, pan edited ND9 and A6 mRNAs were upregulated. The transcript-specific outcomes were also apparent for never-edited transcripts that either remained relatively steady (ND1, ND4 and ND5) or decreased (CO1 and MURF1). Finally, mitochondrial rRNAs remained virtually unaffected indicating an mRNA-specific KPAF5 function (Figure 2C). We note that qRT-PCR detects not only full-length mRNAs but all amplicon-containing species, including primary RNAs, processing and degradation intermediates, and partially-edited molecules. It is possible that some partially-edited or misedited transcripts are stabilized in the absence of a quality surveillance system involving KPAF4/5 complex, which would explain accumulation of ND9 and A6 edited species.

### KPAF4/5 depletion exerts stage-specific effects on mRNA 3′ modification and abundance

**Figure 3.**
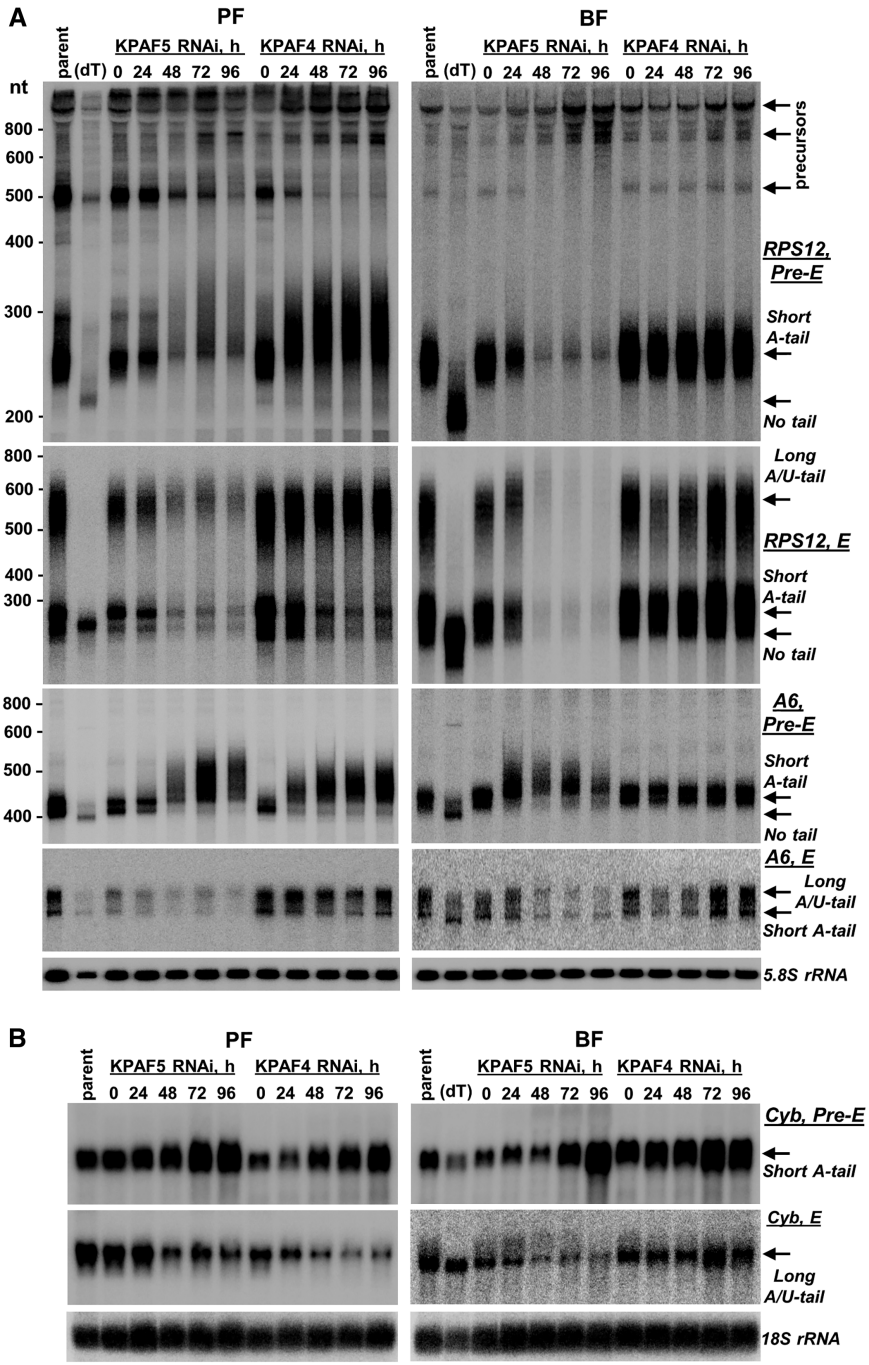
Stage-specific effects of KPAF4 and KPAF5 knockdowns on pan-edited and moderately-edited mRNAs. (**A**) Northern blotting of pre-edited (Pre-E) and fully-edited (E) RPS12 and A6 mRNA variants. Total RNA was separated on a 5% polyacrylamide/8M urea gel and sequentially hybridized with radiolabeled DNA probes. Parent: Lister 427 29-13 strain; (dT), total RNA from parental cell line was hybridized with 20-mer oligo(dT) and treated with RNase H to locate non-adenylated molecules. Zero time point: mock-induced RNAi cell line. Cytosolic 5.8S rRNA was used as loading control. (**B**) Northern blotting of moderately-edited Cyb mRNA. Total RNA was separated on a 1.7% agarose/formaldehyde gel and hybridized with oligonucleotide probes for pre-edited and fully-edited sequences. Loading control: cytosolic 18S rRNA.

**Figure 4.**
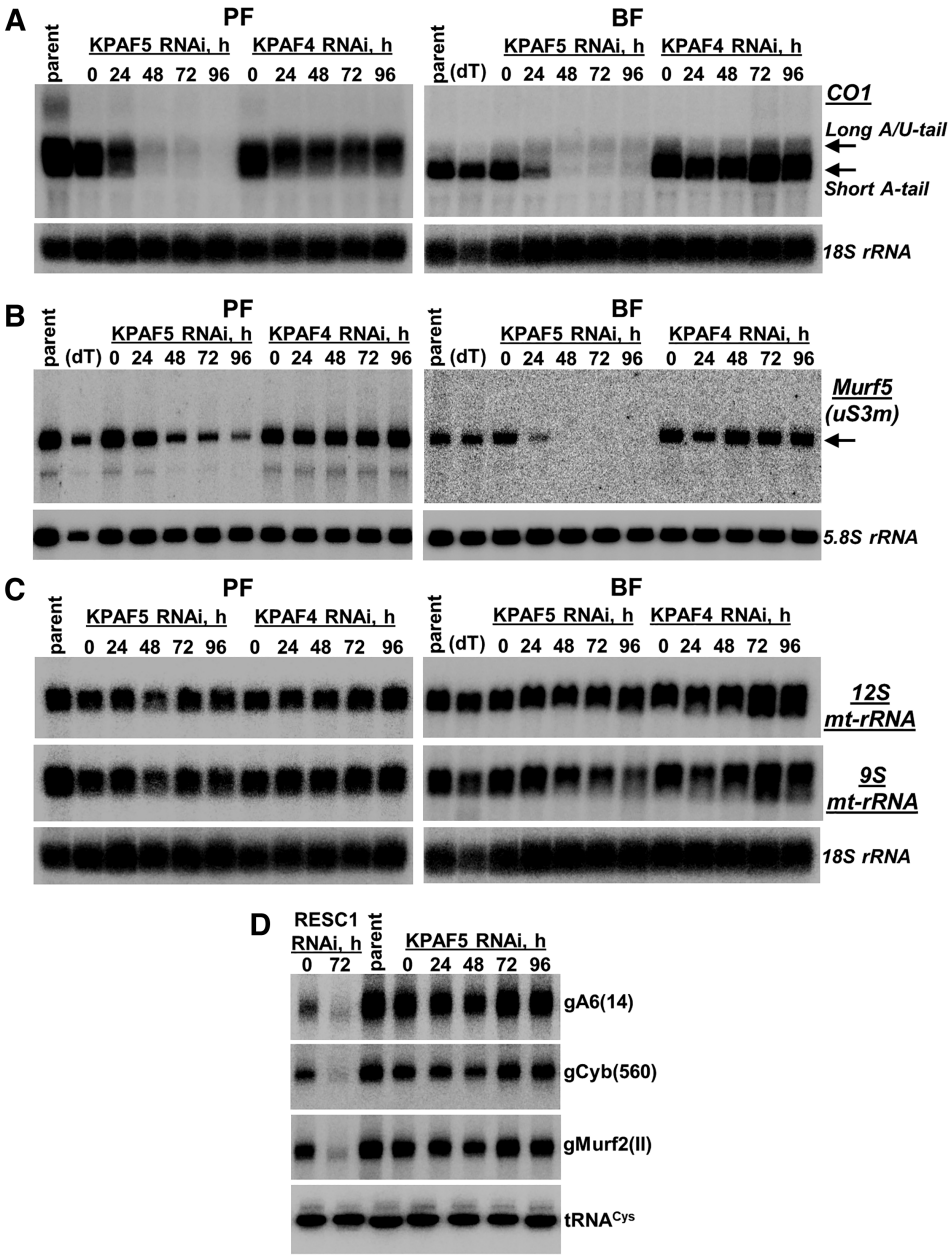
Stage-specific effects of KPAF4 and KPAF5 knockdowns on never-edited mRNAs, ribosomal and guide RNAs. (**A**) Northern blotting of never-edited CO1 mRNA. Total RNA was separated on a 1.7% agarose/formaldehyde gel and sequentially hybridized with oligonucleotide probes. Loading control: cytosolic 18S rRNA. (**B**) Northern blotting of never-edited Murf5 (uS3m) mRNA. Total RNA was separated on 5% polyacrylamide/8M urea gel and hybridized with radiolabeled DNA probe. Loading control: cytosolic 5.8S rRNA. (**C**) Northern blotting of mitochondrial rRNAs. Total RNA was separated on a 1.7% agarose/formaldehyde gel and hybridized with oligonucleotide probes for 9S and 12S rRNAs. Loading control: cytosolic 18S rRNA. (**D**) Guide RNA northern blotting. Total RNA was separated on a 10% polyacrylamide/8M urea gel and sequentially hybridized with oligonucleotide probes specific for minicircle-encoded gA6(14) and gCyb(560) gRNAs that participate in editing of pan-edited and moderately-edited mRNAs, respectively. Maxicircle-encoded gMurf2(II) gRNA was detected on the same membrane. Samples from RNAi cell line targeting the gRNA binding and stabilizing factor RESC1 (GRBC1, GAP2) typify gRNA loss, which causes inhibition of editing (37,47). Mitochondrially-localized tRNA^Cys^ served as loading control.

The changes in relative levels measured by qRT-PCR are instructive of the global transcript abundance but provide limited information about 3′ modifications and their correlation with mRNA editing status. To assess whether KPAF4 and KPAF5 knockdowns induce similar or differential outcomes, we performed time-resolved analysis of pan-edited, moderately-edited, and never-edited mRNAs in procyclic and bloodstream forms by northern blotting. This approach not only quantitatively detects pre-edited and fully-edited variants but also distinguishes non-adenylated, A-tailed and A/U-tailed molecules (Figure 3A). Representative pan-edited mRNAs encoding ribosomal protein RPS12 (uS12m) and ATP synthase subunit 6 (A6) contain a single editing domain in which sequence changes directed by multiple gRNAs commence close to the polyadenylation site and traverse the entire transcript in a 3′-5′ hierarchical order (33). Notably, both encoded proteins are expected to be essential for mitochondrial homeostasis in PF and BF (34,35). As reported for KPAF4 repression in PF (13), U-tailing of adenylated pre-edited mRNA leads to lengthening and a moderate increase in abundance while the fully-edited transcript displays a distinct pattern: the A-tailed form declines while the A/U-tailed form remains unaffected (Figure 3A and Supplementary Table S2). It appears that qRT-PCR analysis detected an increase in partially-degraded edited A6 mRNA species (Figure 2C) whereas fully edited A-tailed and A/U-tailed variants declined. In BF, however, KPAF4 RNAi left all detectable mRNAs virtually unaffected. Conversely, KPAF5 knockdown caused initial lengthening but ultimately degradation of pre-edited transcripts and loss of pan-edited variants in both developmental forms, which is consistent with strong growth inhibition phenotypes (Figure 2A). In moderately-edited Cyb mRNA, where 34 uridines are inserted close to the 5′ end, the pre-edited form was upregulated, but the edited transcript declined in both KPAF4 and KPAF5 RNAi backgrounds (Figure 3B). The Cyb editing is developmentally downregulated in BF (36), but the residual edited mRNA was either negatively impacted by KPAF5 RNAi or unaffected by KPAF4 repression. The never-edited mRNAs, such as CO1 (Figure 4A) and Murf5 (ribosomal protein uS3m, Figure 4B), uniformly declined upon KPAF5 depletion in PF and BF, but were largely unaffected by the loss of KPAF4 in either form. It appears that Murf5 lacks the A-tail detectable by RNase H-oligo[dT] assay but nonetheless follows the uniform downward trend displayed by other mRNAs in KPAF5 knockdown. Finally, the persistence of maxicircle-encoded rRNAs (Figure 4C) and maxicircle- and minicircle-derived gRNAs (Figure 4D) confirmed that KPAF5 is an mRNA-specific factor. Although KPAF5 RNAi effects were more pronounced than those of KPAF4, these observations along with stable KPAF4/5 complex formation are consistent with both proteins acting in the same pathway. We also note differential impacts of KPAF4/5 knockdowns on pre-edited mRNAs that subsequently undergo massive (Figure 3A) or limited (Figure 3B) editing.

### KPAF5 is required for KPAF4 maintenance but not for mRNA adenylation

**Figure 5.**
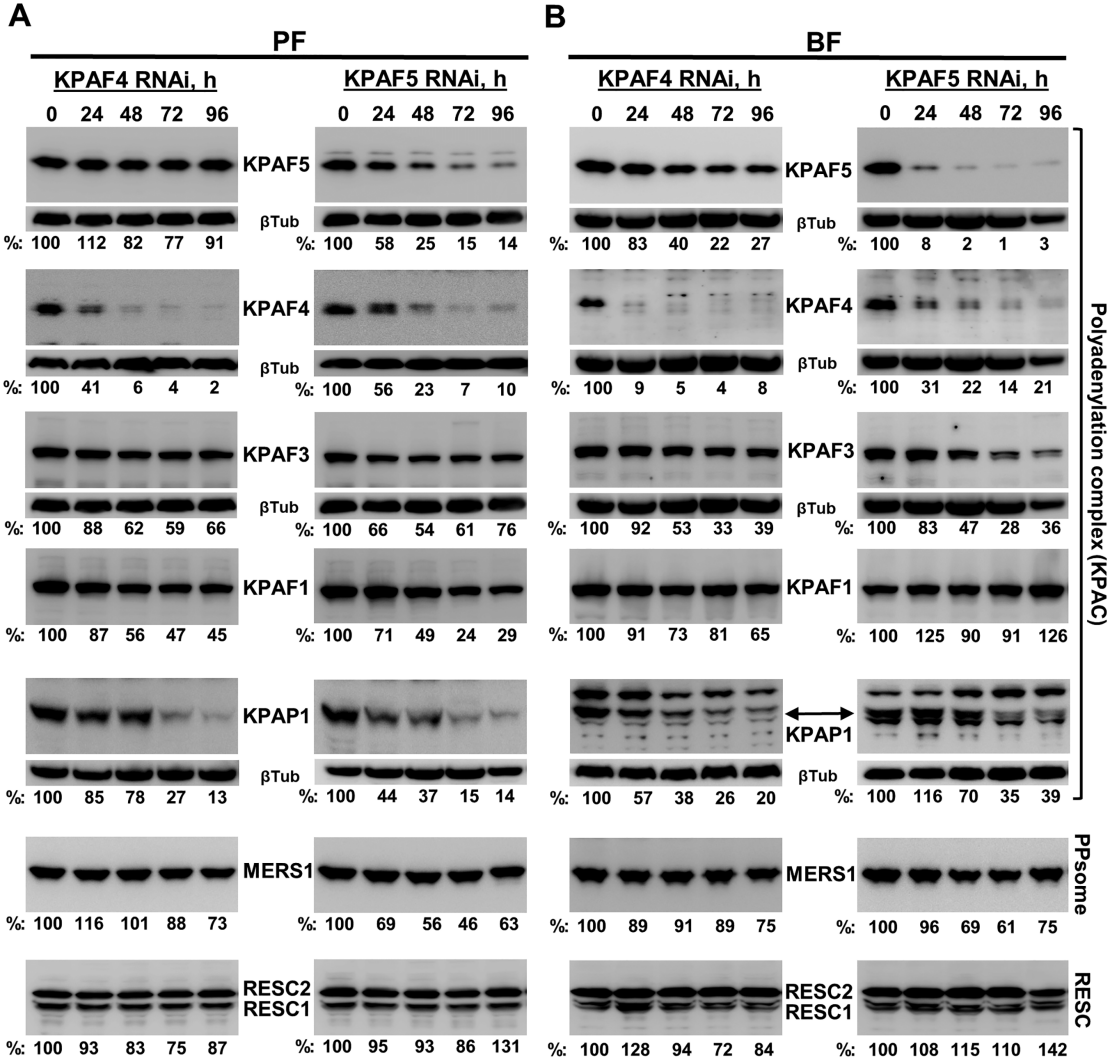
Impact of KPAF4/5 repression on polyadenylation, PPsome, and RESC complex components. Cells collected at indicated KPAF4 and KPAF5 RNAi time points in procyclic (**A**) and bloodstream (**B**) forms were lysed, separated on 8–16% SDS gel, and sequentially probed by quantitative immunoblotting in a top-to-bottom order. Antigen-purified rabbit polyclonal antibodies against KPAF4 and KPAF5 (this study), KPAF1 (12), KPAF3 (6), KPAP1 (10), MERS1 (5) and RESC1/2 (39) were generated in-house against respective recombinant proteins. Signals were normalized to β-tubulin.

KPAF4 and KPAF5 form a stable interaction and likely bind the A-tail as a complex (Figure 1). Since KPAF5 lacks known RNA binding motifs, we next inquired whether KPAF5 may stabilize KPAF4, the RNA binding PPR subunit (13). Immunoblotting with antibodies against components of the KPAC (KPAP1 poly(A) polymerase, KPAF1 adenylation/uridylation and KPAF3 mRNA stabilization factors) demonstrated that indeed KPAF5 repression severely decreases KPAF4 cellular levels in PF and BF developmental forms (Figure 5). However, reciprocal experiments revealed unaltered KPAF5 levels in the KPAF4 RNAi background. These key provisions have been confirmed by conditional PF knockout. In a KPAF5 null background, downregulation of the ectopically-expressed KPAF5 severely reduced KPAF4 and KPAP1 cellular levels (Supplementary Figure S2). Although KPAF4 and KPAF5 knockdowns led to KPAP1 and KPAF1 downregulation in PF, in BF KPAP1 also declined but KPAF1 remained virtually unaffected. Along the same lines, KPAF3 was downregulated by KPAF5 RNAi in BF. The RESC components responsible for gRNA stabilization, RESC1 and RESC2 (37), and the PPsome's catalytic subunit MERS1 remained largely unaffected (5).

**Figure 6.**
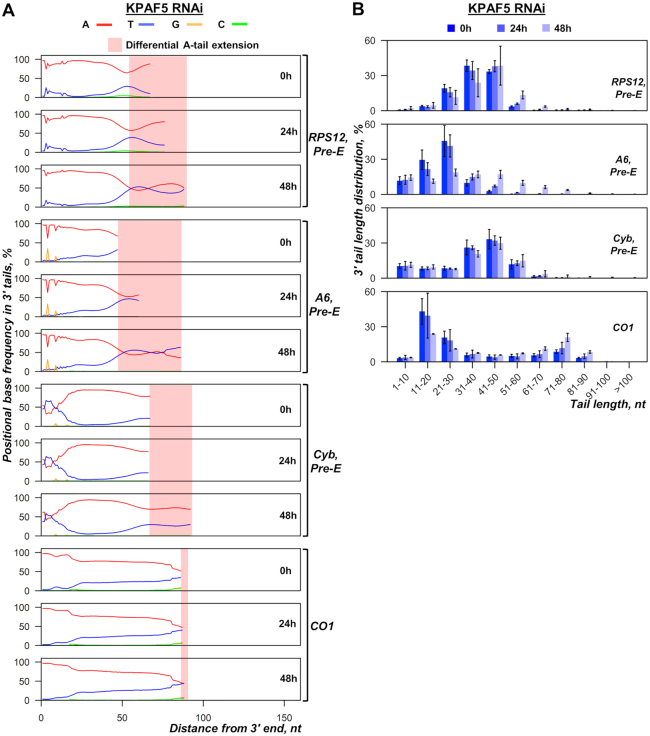
Messenger RNA 3′ modifications in KPAF5 RNAi knockdown. (**A**) Positional nucleotide frequencies in mRNA 3′ extensions. A nucleotide percentage was calculated for each position downstream from the encoded 3′ end set as zero. KPAF5 RNAi was induced for indicated time periods (mean of three biological replicates). The nucleotide bases are color-coded, and the areas of differential A-tail extensions are indicated for pre-edited and never-edited mRNAs. (**B**) Length distribution of mRNA 3′ tails. Non-encoded 3′ end extensions were binned into 10-nt length groups. Relative ratios at 0, 24 and 48 h of RNAi induction are shown as percentage of the total number of reads.

To test whether KPAP1 decline upon KPAF4/5 complex depletion compromised mRNA adenylation and to determine the cause of pre-edited mRNA lengthening prior to degradation (Figure 3A), we sequenced 3′ extensions in pan-edited, moderately-edited and never-edited transcripts (Figure 6). The 3′ extensions were amplified by cRT-PCR from PF at early KPAF5 RNAi time points (23) and sequenced on an Illumina platform in 150 bp pair-end mode. At 48 h post-RNAi induction, the impact KPAF5 and KPAP1 levels and mRNA decline were already apparent, but growth rate remained unaffected (Figure 2A). Figure 6A shows that short A-tails in pre-edited RPS12 and A6 mRNAs become gradually longer with KPAF5 RNAi progression because of increased uridylation of A-tailed species, which is consistent with reported KPAF4 knockdown effects (13). Parsing 3′ extensions into 10 nt bins and plotting each group by RNAi time point also indicated an overall lengthening of A/U-tails beyond 60 nt, which is consistent with intensified uridylation (Figure 6B). Thus, it appears that the loss of KPAF4/5 complex leads to uridylation of short A-tails and earlier emergence of A/U-tails. In aggregate, these data suggest that KPAF4/5 heterodimer acts as a poly(A) binding complex to stabilize adenylated mRNAs and prevent their spurious uridylation. The latter may contribute to premature A/U-tailing and translational activation of partially-edited mRNAs (12).

### Polyadenylation complex binds to both mRNA termini

**Figure 7.**
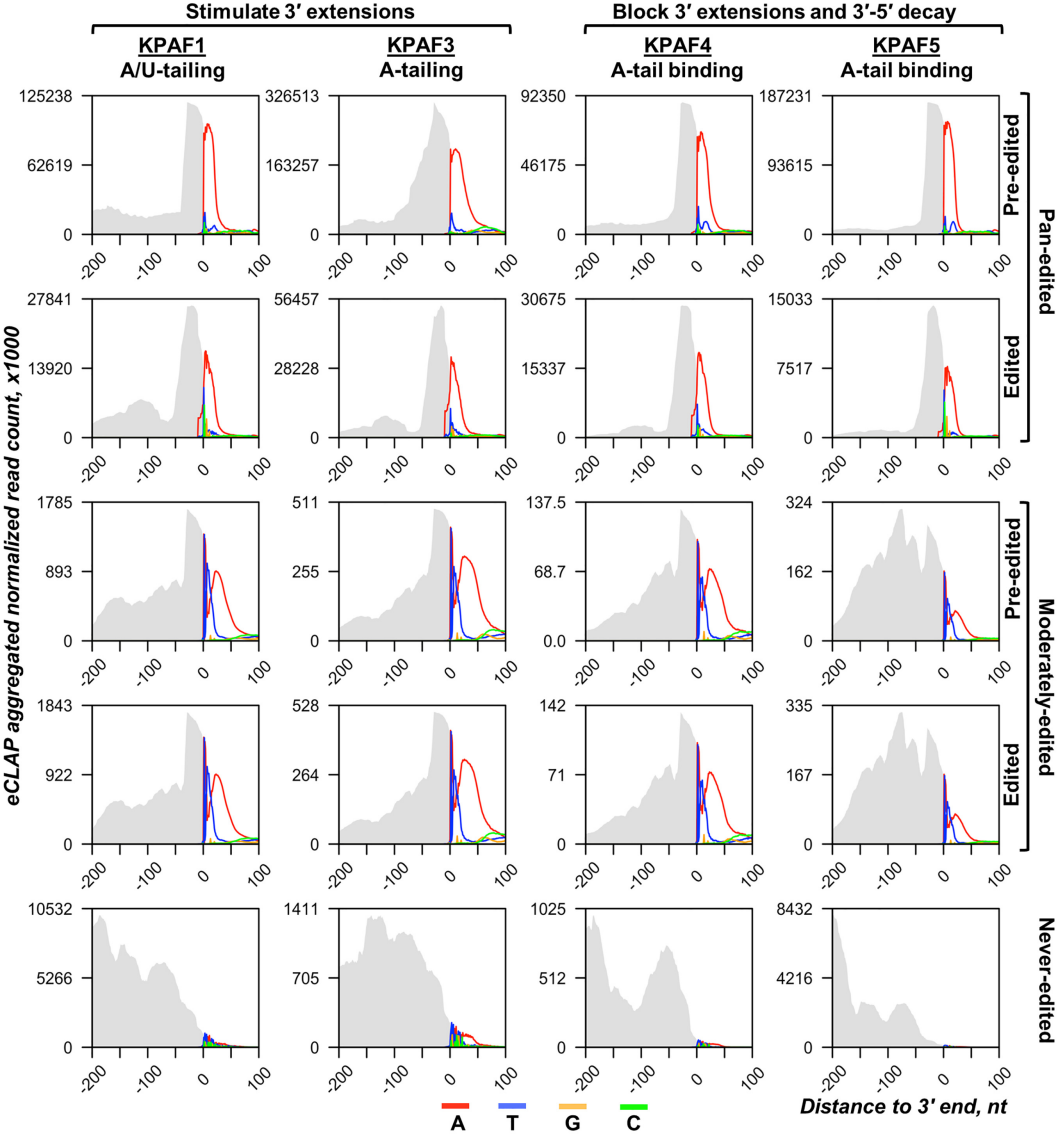
*In vivo* positioning of individual polyadenylation factors in 3′ mRNA regions. The normalized aggregated eCLAP read coverage for each KPAF is represented by the grey area, and the non-encoded nucleotides in 3′ extensions are color-coded. The nucleotide frequency was calculated for each position 200 nt upstream and 100 nt downstream from the mature 3′ end, which was set as zero. The never-edited mRNAs include MURF5, ND1 and CO1; pan-edited profiles include RPS12, A6, ND3, ND7, ND9, CO3, CR3 and CR4; and moderately-edited combine Cyb and Murf2.

To identify KPAF4/5 *in vivo* binding sites and to equate those with KPAF1 and KPAF3 mRNA occupancies, we combined UV-crosslinking in live cells with two-step affinity purification and deep sequencing (eCLAP-Seq). To distinguish RNAs bound to individual subunits in a stable complex, the second purification step (metal affinity) was performed under denaturing conditions, while adapting the eCLIP protocol (38) enabled mapping of RNA–protein crosslinking sites with a single-nucleotide resolution. To account for sequence heterogeneity introduced by editing and 3′ modifications, we incorporated the nucleotide frequency within reads that partially mapped to edited transcripts or to 3′ UTR-tail junctions, thus extending coverage beyond encoded 3′ regions (5,29). This produced an aggregated plot with mapped reads shown as grey area and color-coded nucleotide frequency. The plot covers 200 nt upstream and 100 nt downstream from the mature 3′ end, which was set as zero for pre-edited, edited and never-edited mRNAs (Figure 7). Targeted tail sequencing, eCLAP and global mitochondrial RNA-Seq statistics are provided in Supplementary Table S3. Irrespective of specific function in mRNA stabilization and 3′ modification, all KPAFs preferentially bind to 3′ UTRs near polyadenylation sites and to short A-tails, but not long A/U-tails. This pattern also applies to KPAF5, the only non-PPR among polyadenylation factors.

**Figure 8.**
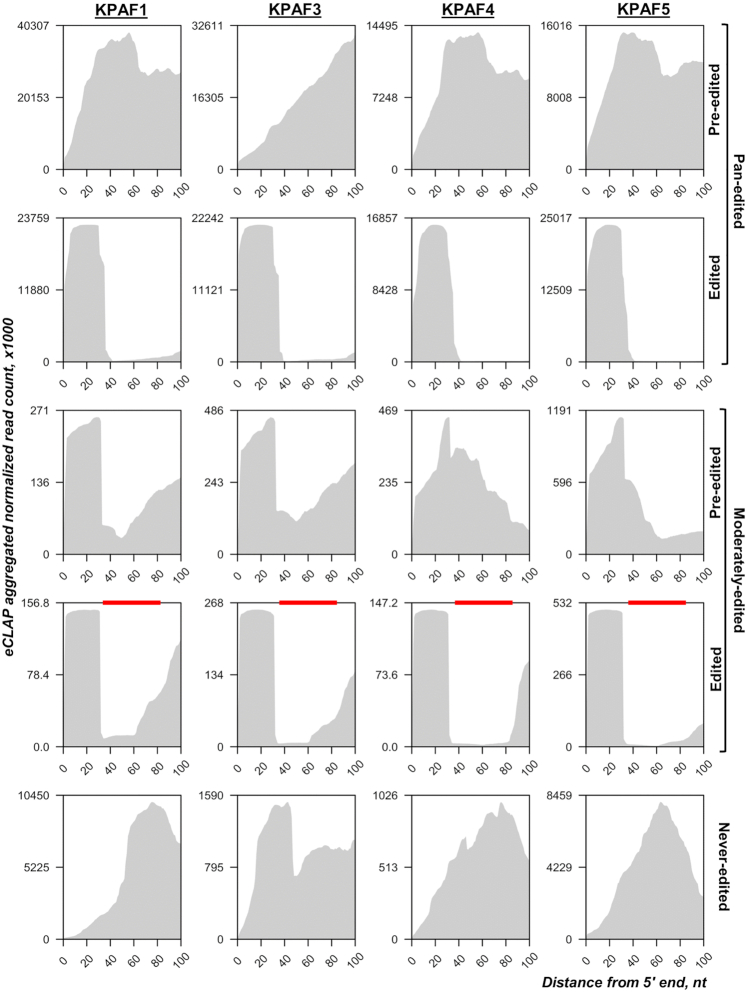
Polyadenylation complex binding to 5′ end regions. The normalized aggregated eCLAP read coverage of the 100 nt downstream from the mature 5′ end is delineated by the grey area. Transcript categories defined as in Figure 7. In moderately-edited mRNAs, the edited domains are shown by red bars.

KPAF5 knockdown leads to mRNA uridylation and degradation but does not interfere with mRNA A-tailing (Figure 6). It seems evident that the A-tail's stabilizing function (10,11) entails binding of this *cis*-element by KPAF4/5, which forms a ribonucleoprotein complex resistant to MPsome assault (13). Conversely, the loss of KPAF4/5 enables mRNA uridylation by the MPsome-embedded KRET1 TUTase and subsequent activation of the MPsome's degradation activity carried out by KDSS1 exonuclease (9). However, the co-purification and *in vivo* proximity networks (Figure 1C) predict extensive interaction among KPAC, RESC, and the PPsome, which preferentially binds to the mRNA 5′ end. This event and subsequent 5′ pyrophosphate hydrolysis are also required for mRNA protection against the MPsome attack (5). Collectively, these observations suggest a crosstalk between 5′ and 3′ ends that is likely mediated by contacts between the PPsome and KPAC occupying the respective termini. To test this hypothesis, we compared eCLAP coverage of a region extending 100 nt downstream from the mature 5′ ends (Figure 8). In pan-edited mRNAs, all KPAFs displayed invariable binding to the 5′ region in a pre-edited transcript, while in edited form the binding sites were retained only in unedited 5′ UTRs, and largely eliminated by contiguous sequence changes in the coding region. This correlation aligns with a critical role played by the A-tail in stabilizing edited mRNA, and its non-essentiality for maintaining pre-edited mRNA (10). In moderately-edited mRNAs, the reads re-distribution between pre-edited and edited forms was also consistent with KPAFs displacement by the editing process, which is limited to regions marked with red bars (Figure 8). These results indicate that KPAF4/5 binds to both the 5′ and 3′ termini in pre-edited and edited transcripts, possibly leading to mRNA circularization. It follows that circularization may impede MPsome access to the 3′ end and thus contribute to mRNA resistance to 3′-5′ degradation (6). Our observations also rationalize rapid mRNA decay in MERS1 knockdown (37). This NUDIX pyrophosphohydrolase binds to the 5′ terminus as a component of the PPsome complex and removes pyrophosphate from the first nucleotide incorporated by transcription. Considering PPsome-KPAC interaction's potential contribution to mRNA circularization, the loss of the former complex may prevent tethering of 5′ and 3′ termini and expose RNA to MPsome attack (5). Thus, it appears that both the PPsome and KPAC are required for mRNA stabilization, but neither is individually sufficient.

### RESC bridges KPAC and the PPsome to stabilize edited and never-edited mRNAs

**Figure 9.**
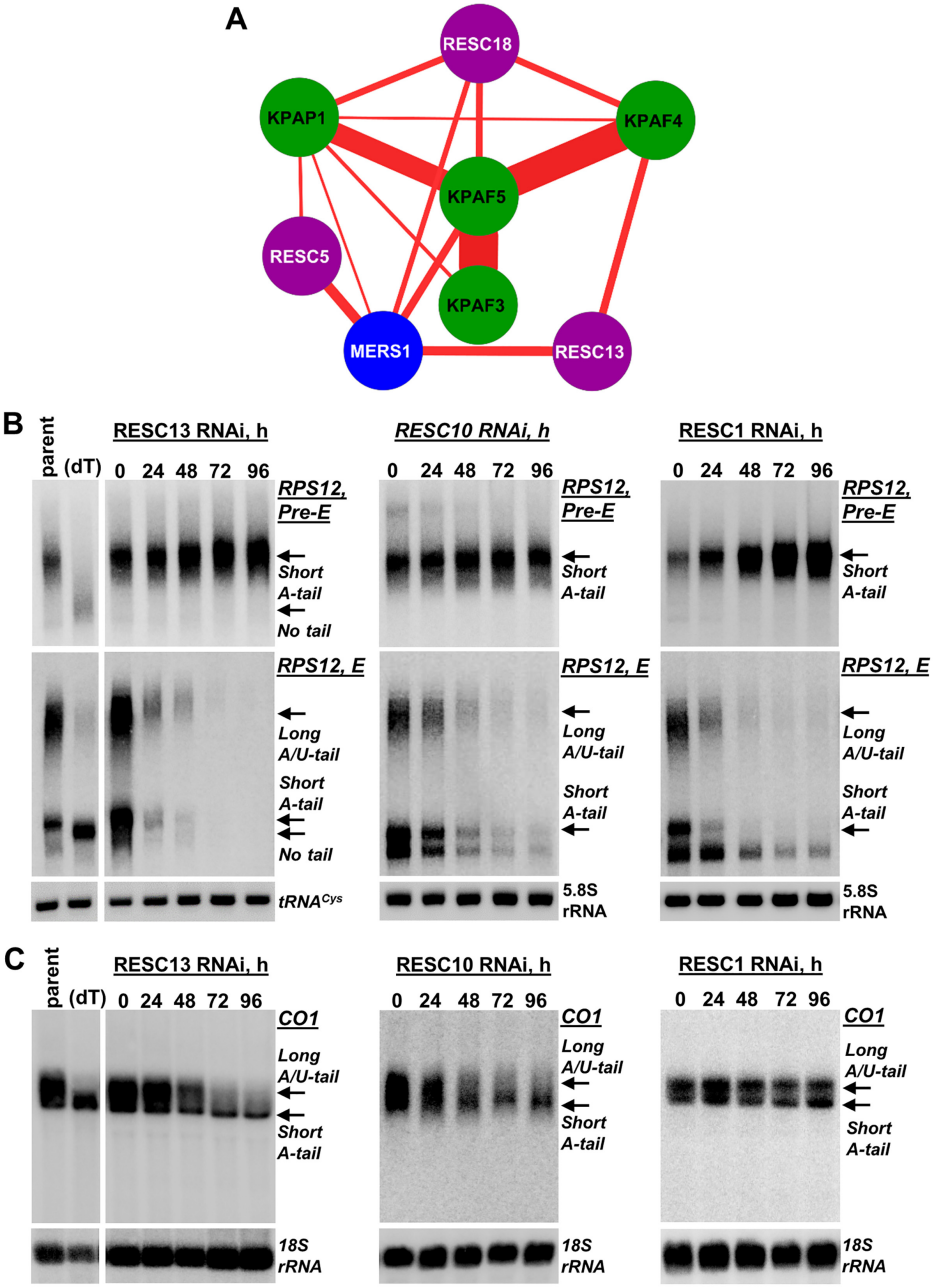
RNA editing substrate binding complex (RESC) stabilizes never-edited mRNAs. (**A**) *In vivo* proximity network of KPAP1 poly(A) polymerase, KPAF3, 4 and 5, and RESC5, 13 (RGG2) and 18, and PPsome's pyrophosphohydrolase MERS1. The BioID experiments were performed in parallel under uniform induction, biotinylation, and purification conditions. The network was generated in Cytoscape software. The edge thickness correlates with normalized spectral abundance factor (NSAF) ranging from 1.13 × 10^−4^ (MERS1–KPAP1) to 1.5 × 10^−3^ (KPAF4–KPAF5) and reflects the predicted interaction strength (Supplementary Table S1). (**B**) Northern blotting of pre-edited (Pre-E) and fully-edited (E) RPS12 mRNA variants in RESC10, RESC13 (RGG2) and RESC1 RNAi knockdowns. Total RNA was separated on a 5% polyacrylamide/8M urea gel and sequentially hybridized with radiolabeled DNA probes. Parent: Lister 427 29–13 strain; (dT), RNA was hybridized with 20-mer oligo(dT) and treated with RNase H to remove A-tails and locate migration positions of a non-adenylated molecules. Short A-tailed and A/U-tailed mRNAs are indicated by arrows. Zero-time point: mock-induced RNAi cell line. Cytosolic 5.8S rRNA or tRNA^Cys^ were used as loading control. (**C**) Northern blotting of never-edited CO1 mRNA in RESC10, RESC13 and RESC1 RNAi knockdowns. Total RNA was separated on a 1.7% agarose/formaldehyde gel and hybridized with oligonucleotide probe. Loading control: 18S rRNA.

Poly(A) binding KPAF4/5 complex predominantly binds mRNA 3′ ends but also shows a substantial occupancy of the 5′ region (Figures 7 and 8). Conversely, KPAF5 knockdown leads to pre-mRNA uridylation and rapid decay in procyclic and bloodstream developmental forms (Figures 3, 4 and 6). These MPsome-catalyzed processes are apparently countered by KPAF4/5 binding to the A-tail, but the contribution of KPAF4/5 interaction with mRNA’s 5′ end is unclear. Likewise, a cohesive mechanism for mRNA stabilization ought to account for the essential role of the 5′ end-bound PPsome complex (5). To elucidate the functional connection between the PPsome and KPAC, we reasoned that since the components of both complexes have been detected in purified RESC, but not in reciprocal purifications (5,37,39), the RESC may tether these 5′end and 3′ end-bound particles. An ensuing mRNA circularization would be an apparent outcome leading to protection against MPsome-catalyzed uridylation and decay. To gain a higher-resolution view of interactions between individual polyadenylation factors and specific RESC and PPsome subunits, we performed BioID experiments with RESC5, RESC13 (RGG2) and RESC18, KPAP1 poly(A) polymerase, KPAF3, KPAF4, KPAF5 and MERS1 BirA* ligase fusion proteins (Figure 9A). The RESC subunits typify modules responsible for interactions with gRNA (RESC5), RECC (RESC13 (RGG2)), and KPAC (RESC18) (39–41). The proximity network depicts KPAF5 as central to KPAC assembly whereby direct interaction between KPAF5 and KPAF4 is comparable to those of KPAF5 with KPAF3 and KPAP1. Indeed, the KPAF5–KPAP1 and KPAF5–KPAF3 predicted contacts indicate a high probability of direct interactions in the assembled KPAC and may explain KPAP1 loss in KPAF5 PF and BF knockdowns, and downregulation of KPAF3 in KPAF5 BF RNAi cells (Figure 5). The lack of appreciable proximity between individual RESC subunits demonstrates high granularity of this approach and supports their belonging to distinct modules within RESC. Remarkably, RESC5 appears to interact with the PPsome component MERS1 while RESC13 shows predicted contacts with both MERS1 and KPAF4. RESC13 is an RGG motif-containing RNA binding protein that stimulates processivity of pan-editing (40,42,43).

The RESC-mediated model of mRNA circularization implies that, in addition to its well-established role in gRNA binding and editing (3,4), this complex may play a critical role in stabilizing never-edited mRNAs, as do PPsome (5) and KPAF4/5 (Figures 2 and 4). To test this hypothesis, we analyzed the impact of RESC13 RNAi knockdown on pre-edited and edited (Figure 9B), and never-edited mRNAs (Figure 9C). In agreement with published data, RESC13 knockdown did not compromise pre-edited mRNA stability but efficiently blocked RPS12 mRNA editing. Likewise, downregulation of the A/U-tailed translationally-competent CO1 mRNA form (11) in RESC13 RNAi confirmed RESC participation in stabilizing never-edited mRNAs. This finding contradicts previously reported 2-fold upregulation of CO1 mRNA in RESC13 knockdown (43), but the discrepancy is likely explained by the detection methods. Quantitative RT-PCR used by Fisk et al. and northern blotting in this study reflect the overall abundance of CO1 mRNA-containing species and full-length mRNA, respectively. To further confirm participation of the RESC13 cluster (3,18,44) in stabilizing never-edited mRNAs, we knocked down RESC10, which presumably belongs to the RESC13 sub-complex (3), and the plausibly remote gRNA binding protein RESC1. To that end, RESC10 RNAi negatively impacted pan-edited and never-edited mRNAs, while RESC1 repression and the ensuing gRNA loss (37) affected only the editing process (Figure 9B, C). Collectively, UV-crosslinking, *in vivo* biotinylation, and RNAi knockdown experiments indicate that RESC-mediated tethering of PPsome and KPAC leads to circularization, which constitutes a mechanistic basis for mRNA resistance to MPsome-catalyzed 3′-5′ decay.

## DISCUSSION

**Figure 10.**
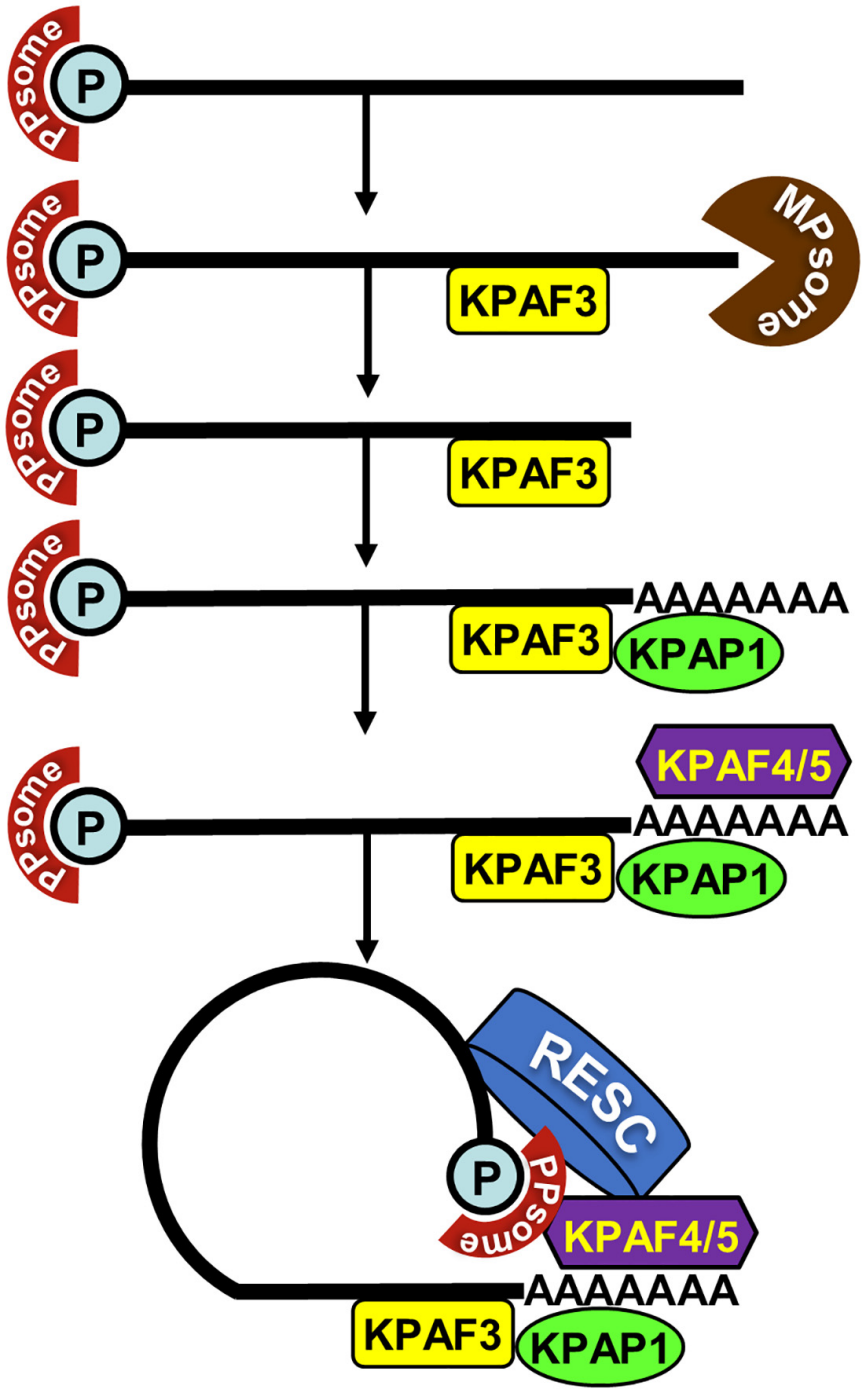
A circularization model of mitochondrial mRNA stabilization pathway in trypanosomes. We propose that mRNA stability is determined by PPsome and polyadenylation complexes that sequester 5′ and 3′ termini, respectively. It appears that distinct modalities within RNA editing substrate binding complex (RESC) interact with these end-bound particles to facilitate mRNA circularization and resistance to 3′-5′ degradation by the MPsome.

This study identifies KPAF5, a novel component of the mitochondrial polyadenylation complex that is essential for the viability of *Trypanosoma brucei* insect and bloodstream forms. We demonstrate that, together with PPR protein KPAF4, this polypeptide lacking known motifs forms a stable KPAF4/5 heterodimer. We present evidence that KPAF4/5 constitutes a poly(A) binding entity within the KPAC and is responsible for critical mRNA stabilizing functions (Figure 10). Polyadenylation's effect on mRNA resistance to 3′-5′ degradation depends on the state of internal U-insertion/deletion editing. Specifically, the A-tail is dispensable for pre-edited mRNA stability but is strictly required for maintaining transcripts edited beyond the 3′ region (10,11). This toggle phenomenon has been attributed to KPAF3 binding to the G-rich pre-edited mRNA 3′ terminus and ensuing KPAP1 recruitment and A-tailing. In the current model, KPAF3 displacement by initial editing events leaves the partially-edited mRNA reliant on the A-tail for protection from MPsome attack (6). Although degradation proceeds in the 3′-5′ direction, mRNA stabilization also requires PPsome binding to the 5′ end (5). We have previously identified KPAF4 as the poly(A) binding factor essential for normal parasite growth and demonstrated its role in preventing uridylation and degradation of edited mRNAs that are no longer protected by KPAF3 (5,13). However, in depth-analysis of KPAF4 interactions by affinity purification, *in vivo* biotinylation, *in vitro* synthesis, and co-expression in bacteria revealed formation of a stable heterodimer with KPAF5 (Figure 1). This interaction is definitively required for maintaining KPAF4 in the cell (Figure 5). Furthermore, the downregulation of KPAP1 poly(A) polymerase and KPAF3 ‘editing sensor’ in KPAF5 knockdown, along with binary KPAP1-KPAP5 and KPAF3-KPAF5 interactions predicted by *in vivo* biotinylation (Figures 1C and 9A), positions KPAF5 as the interactions core of the polyadenylation complex. Although *in vivo* UV-crosslinking experiments provide evidence of KPAF5-RNA contacts (Figures 7 and 8), the chief reason for this protein's essentiality in PF and BF appears to be participation in a heterodimer that stabilizes poly(A) binding PPR factor KPAF4 (Figure 5 and Supplementary Figure S3).

Systematic mapping of kinetoplast polyadenylation factors’ *in vivo* binding sites revealed that, in addition to expected recognition of the 3′ region and the A-tail (Figure 7), KPAFs also bind throughout pre-edited and never-edited transcripts (Figure 8 and Supplementary Figure S4–S7). The editing process, whether by virtue of sequence changes or ribonucleoprotein complex remodeling, displaces KPAFs from much of the coding region (pan-edited RNAs), or from a limited segment (moderately-edited RNAs). However, the 5′ UTRs remain bound by KPAFs irrespective of mRNA’s editing status. Thus, a consistent coverage of 5′ and 3′ UTRs and the A-tail by KPAFs substantiates a conclusion that mRNAs termini are maintained in proximity. This raises the question whether such tethering involves direct or mediated PPsome-KPAC interaction.

The RNA editing substrate binding complex (RESC) has recently emerged as a multifunctional platform that binds RNA editing substrates (pre-edited mRNAs and gRNAs), intermediates (partially-edited transcripts) and edited products (4). Accordingly, RESC co-purifies with sub-stoichiometric amounts of RNA editing catalytic complex (RECC), the PPsome (5,37) and KPAC (13,39). These findings suggest that RESC functions extend beyond editing and may include stabilization of never-edited mRNAs. It stands to reason that the RESC-dependent mechanism of protecting never-edited mRNA may involve both 5′ end-bound PPsome and 3′ end-bound KPAC. In this context, our study provides evidence of *in vivo* proximity between distinct RESC subunits and components of these complexes (Figures 1C and 9A). Furthermore, we show that RESC13 (RGG2), a subunit previously implicated in promoting processivity of editing in pan-edited transcripts, and RESC10 are essential for never-edited mRNA stability. In agreement with interaction assessments by yeast two-hybrid (40) and co-purification (39) approaches, RESC13 and RESC10 belong to the same sub-complex, and it seems plausible that their knockdowns exert similar effects. Conversely, repression of the gRNA-stabilizing subunit RESC1 selectively eliminates gRNAs and inhibits the editing process (37) but leaves never-edited mRNAs unaffected (Figure 9B, C). These findings underscore distinct functionalities of RESC modules, among which the RESC13-containing cluster is apparently critical for mRNA stabilization irrespective of its editing status. Overall, it appears that interactions among distinct modules within RESC (39), the PPsome, and KPAC manifest in mRNA circularization. This may constitute a mechanistic link between 5′ pyrophosphate removal and 3′ adenylation in stabilizing edited and never-edited mRNAs.

## Supplementary Material

gkaa575_Supplemental_Files
