# Supplementary material for: Poly(A) binding KPAF4/5 complex stabilizes kinetoplast mRNAs in Trypanosoma brucei
Source: Nucleic Acids Res. 2020 Jul 2;48(15):8645–62. doi: 10.1093/nar/gkaa575 (PMC7470953; doi:10.1093/nar/gkaa575)
Supplement: gkaa575_Supplemental_Files [file gkaa575_supplemental_files.zip › Supp_Info_NAR_2020_Final.pdf]

Supplementary information

Poly(A) binding KPAF4/5 complex stabilizes kinetoplast mRNAs in *Trypanosoma brucei*

Inna Aphasizheva[1], Tian Yu[1], Takuma Suematsu[1], Qiushi Liu[1], Mikhail V. Mesitov[1], Clinton Yu[2], Lan Huang[2], Liye Zhang[3] and Ruslan Aphasizhev[1,4*]

[1]Department of Molecular and Cell Biology, Boston University Medical Campus, Boston, MA 02118, USA

[2]Department of Physiology and Biophysics, School of Medicine, University of California, Irvine, CA 92697, USA

[3]School of Life Science and Technology, ShanghaiTech University, Shanghai 201210, China

[4]Department of Biochemistry, Boston University Medical Campus, Boston, MA 02118, USA

***Corresponding author:** Ruslan Aphasizhev, Department of Molecular and Cell Biology, 72 E. Concord St., E424, Boston, MA 02118, USA

Email: ruslana@bu.edu; **Fax:** 617-414-1056; **Phone:** 617-358-3773.

https://orcid.org/0000-0002-4595-6618

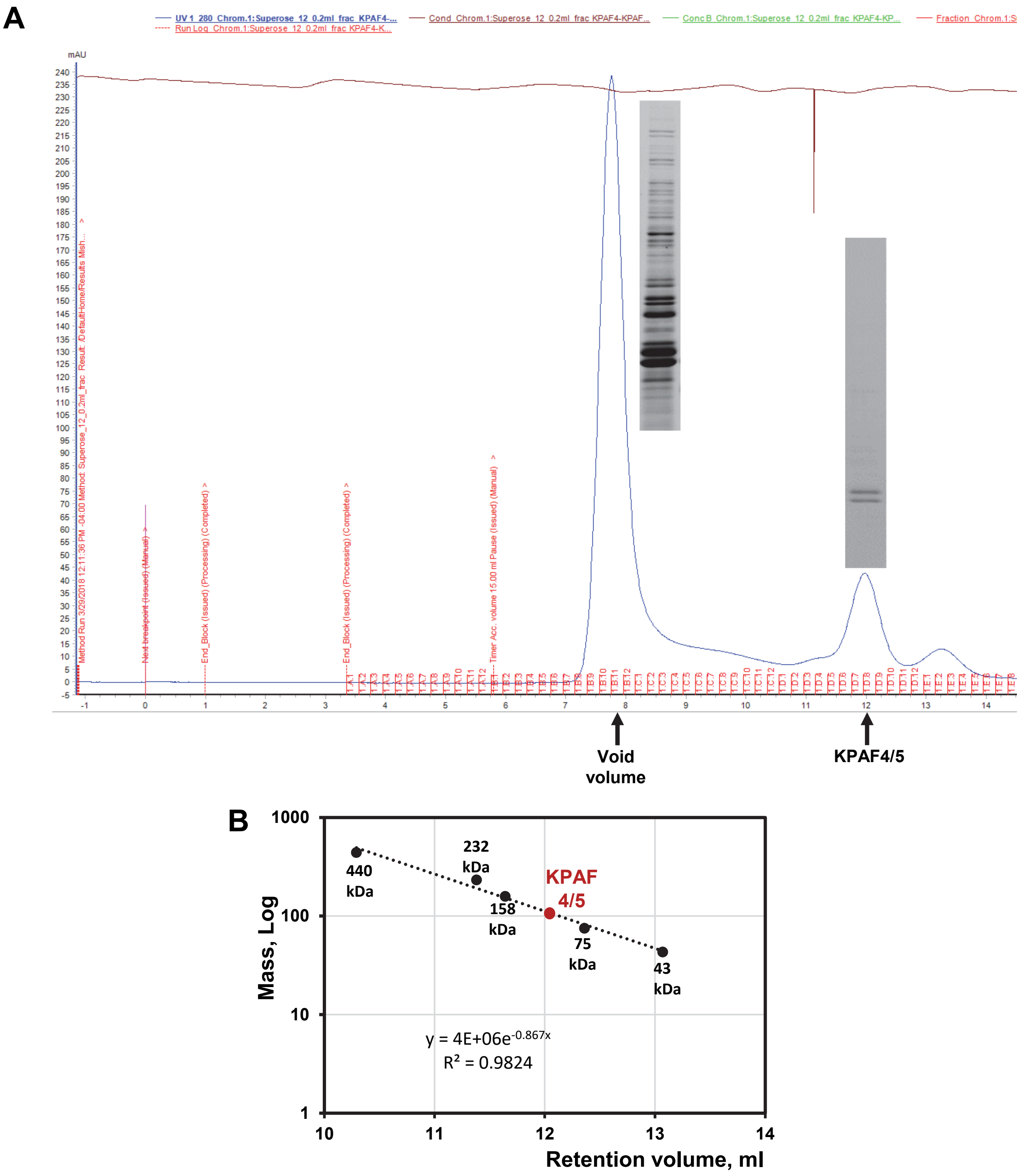

Supplementary Figure S1

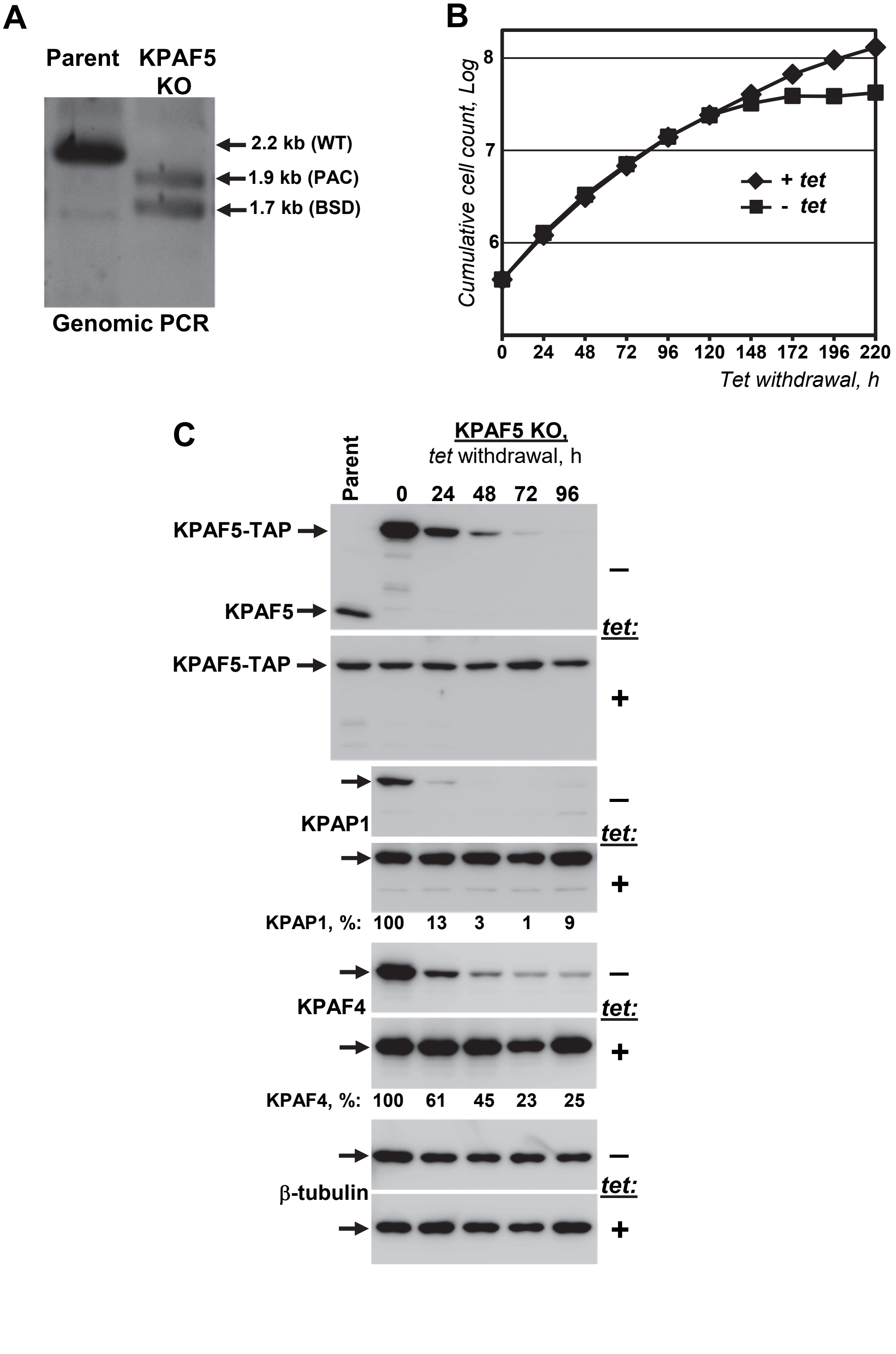

Supplementary Figure S2

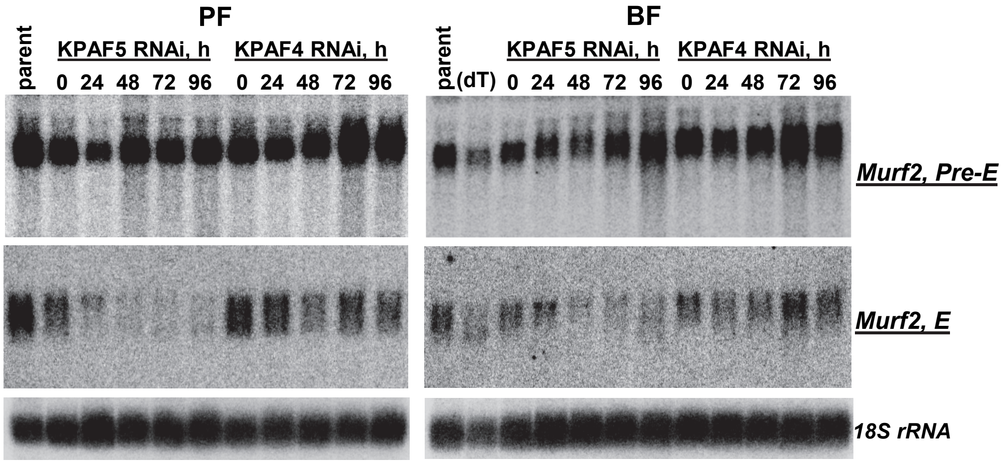

Supplementary Figure S3

KPAF1 eCLAP

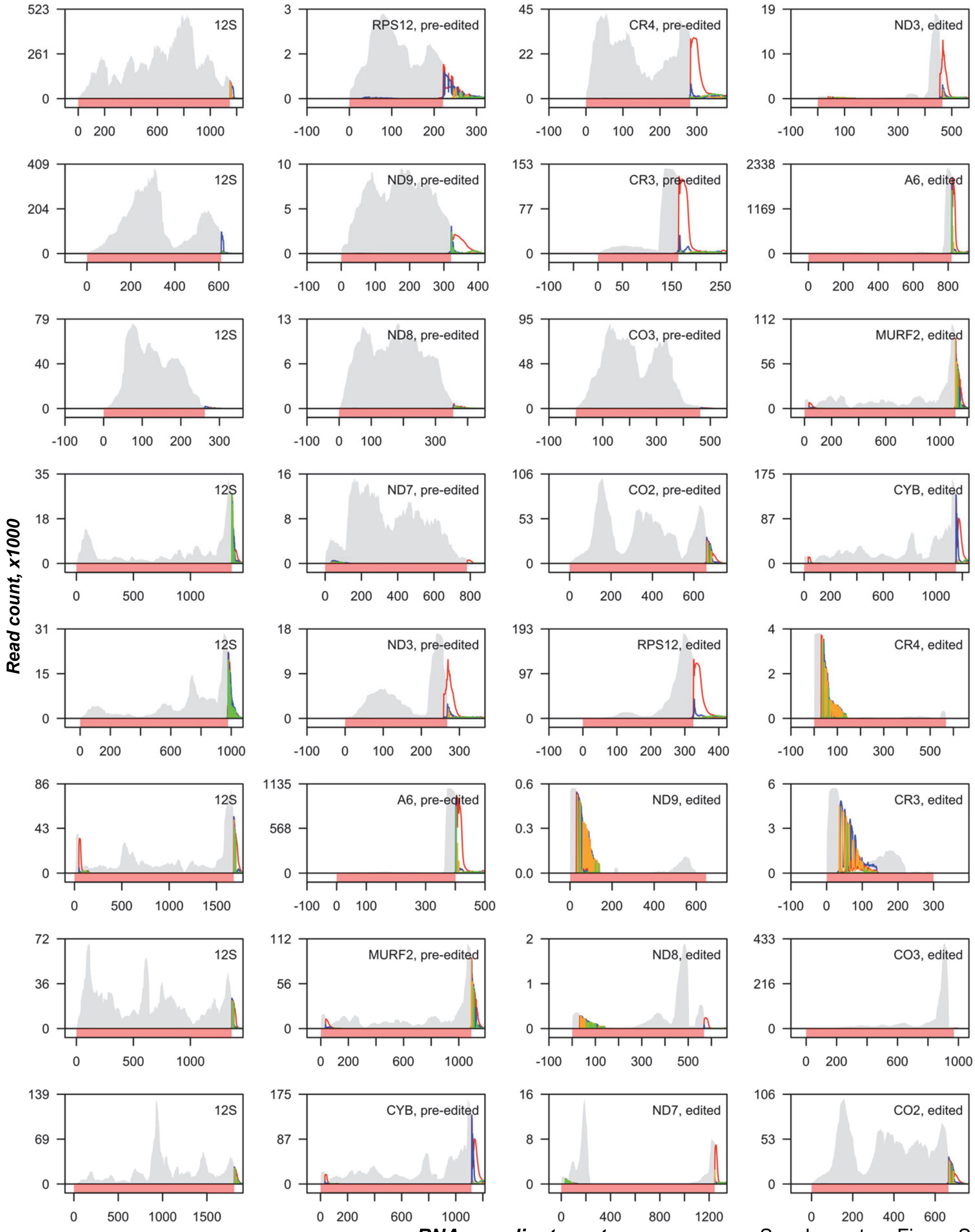

Supplementary Figure S4

KPAF3 eCLAP

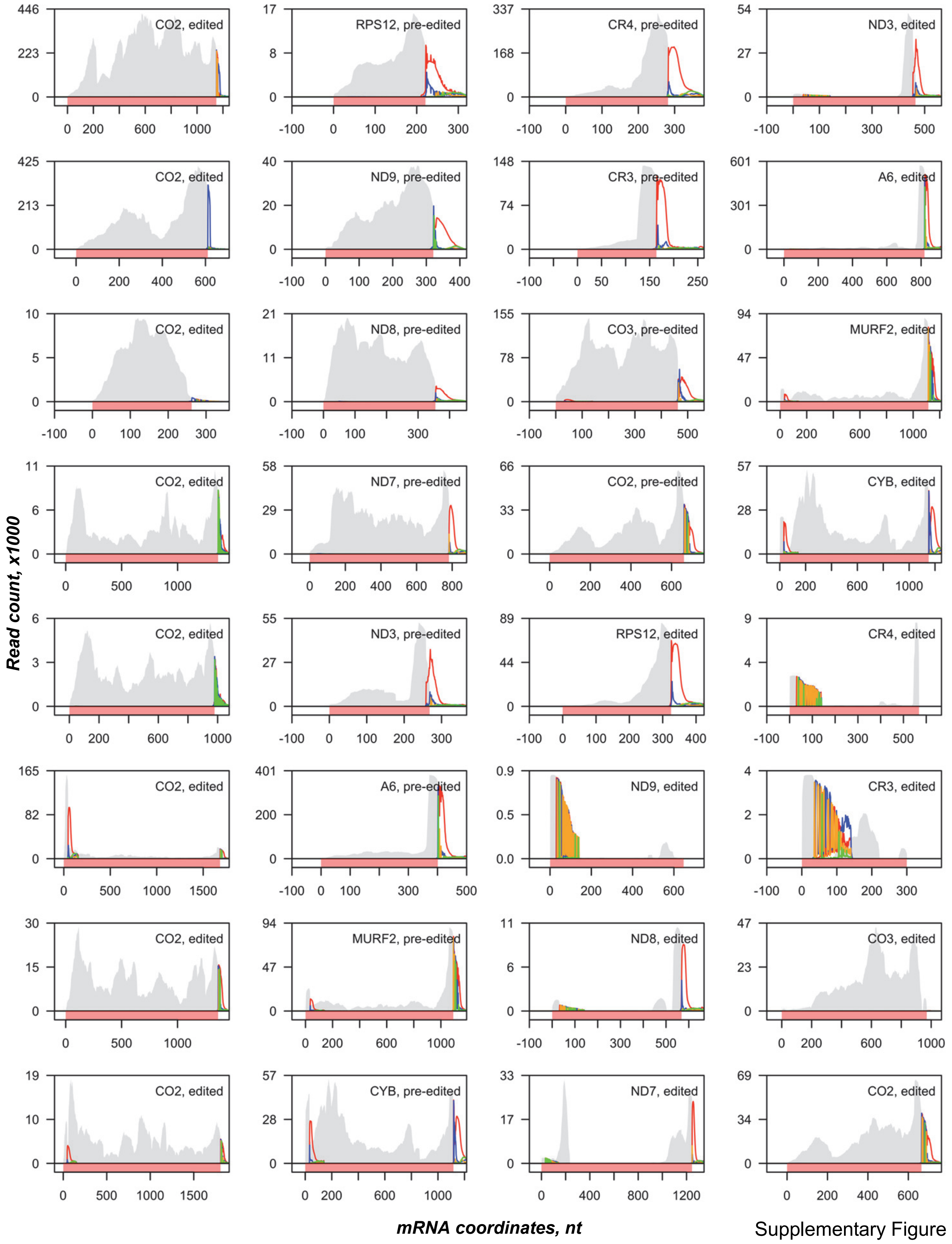

Supplementary Figure S5

KPAF4 eCLAP

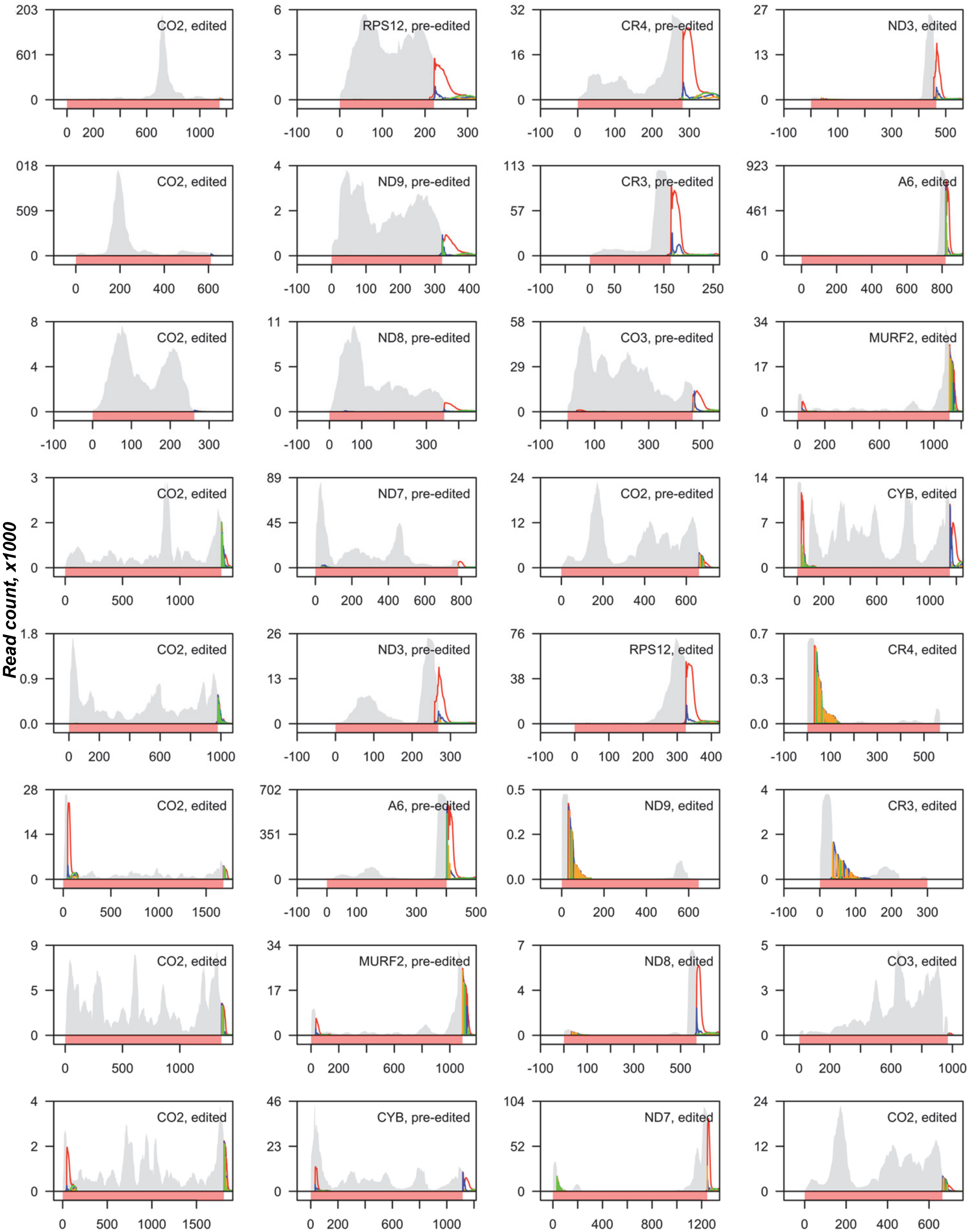

Supplementary Figure S6

KPAF5 eCLAP

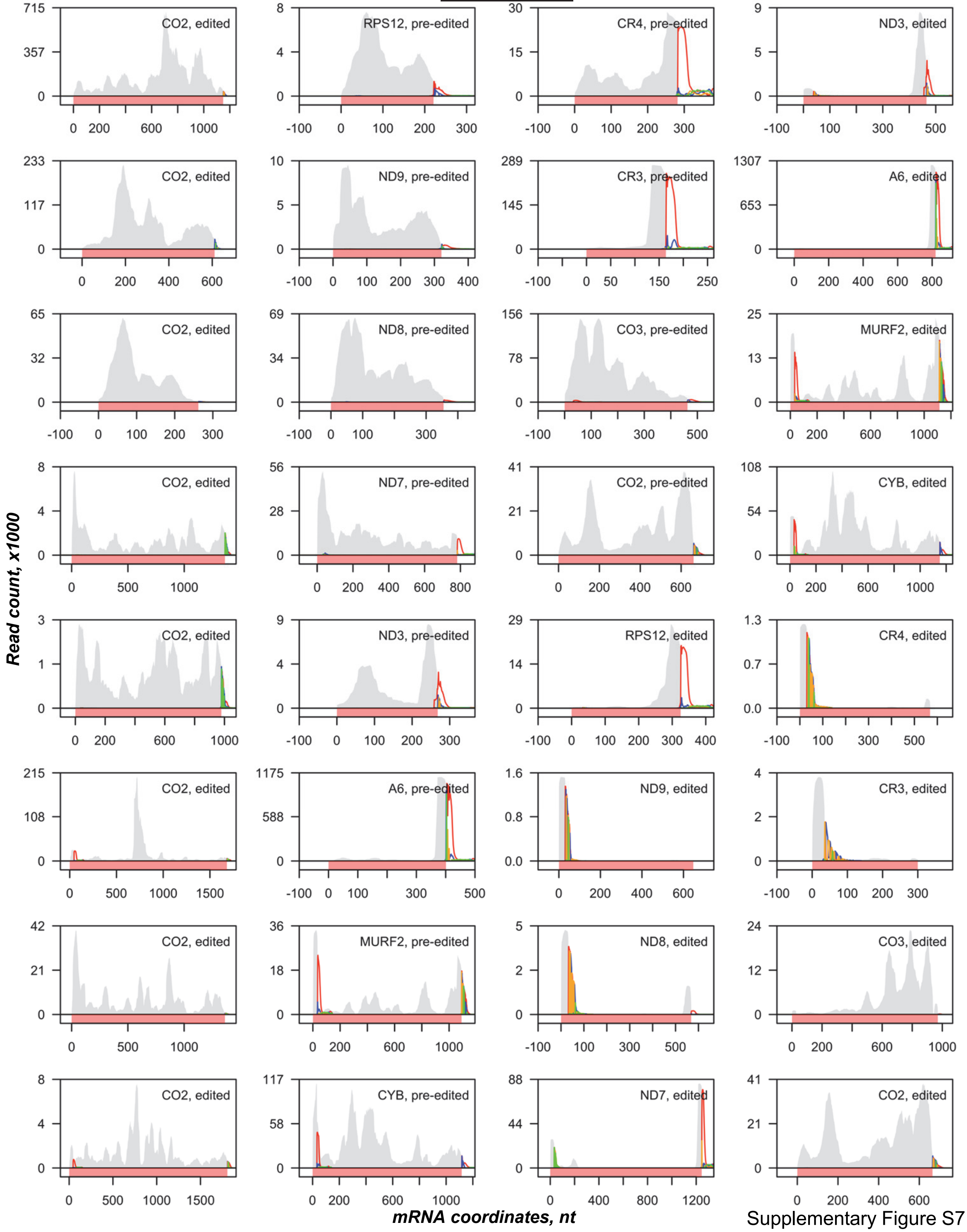

Supplementary Figure S7

Supplementary figures and tables legends

Supplementary Figure S1. Apparent molecular mass of KPAF4/5 complex purified from *E. coli.* (**A**) Size exclusion chromatographic profile. Concentrated fraction from Strep Tactin affinity column was separated on Superose 12 10/300 GL column (GE Healthcare). Void and KPAF4/5 elution volumes are indicated by arrows. Insets show protein profiles of fractions corresponding to major peaks of OD^{280}. (**B**) Superose 12 10/300 GL column calibration. Molecular mass standards (GE Healthcare: Ferritin, 440 kDa; Catalase, 232 kDa; Aldolase, 158 kDa; Albumin, 75 kDa and Ovalbumin, 43 kDa) were separated in 20 mM HEPES, pH 7.5, 150 mM KCl, 1 mM DTT at 0.2 ml/min. KPAF4/5 eluted at apparent molecular mass of ~90 kDa.

Supplementary Figure S2. Conditional KPAF5 knockout in PF. **(A)** Elimination of both endogenous alleles and insertion of antibiotic resistance genes was verified by genomic PCR. **(B)** Cell growth inhibition after suppression of the ectopically expressed KPAF5. In the absence of tetracycline, *tet* repressor re-binds to *tet*-operator and inhibits TAP-tagged KPAF5 expression from the rRNA locus. **(C)** Quantitative immunoblotting of endogenous and TAP-tagged KPAF5, KPAP1 poly(A) polymerase and KPAF4 PPR in parasite cells collected at indicated times. Change in relative abundance was calculated with β-tubulin as loading control.

Supplementary Figure S3. Northern blotting of moderately-edited mRNA in KPAF4 and KPAF5 RNAi knockdown cell lines. Total RNA was separated on a 1.7% agarose/formaldehyde gel and sequentially hybridized with oligonucleotide probes. Loading control: cytosolic 18S rRNA.

Supplementary Figure S4. KPAF1 eCLAP read mapping to mitochondrial mRNAs.

Supplementary Figure S5. KPAF3 eCLAP read mapping to mitochondrial mRNAs.

Supplementary Figure S6. KPAF4 eCLAP read mapping to mitochondrial mRNAs.

Supplementary Figure S7. KPAF5 eCLAP read mapping to mitochondrial mRNAs.

Supplementary Table S1. LC-MS/MS analysis of tryptic peptide samples from co-purification (rapid pulldown of TAP-tagged proteins) and BioID (proximity biotinylation with BirA* fusion proteins) experiments. Raw data and files used to build interaction networks are listed for each bait protein as separate sheets.

Supplementary Table S2. Quantitation of northern blotting experiments. Developmental forms, RNAi cell lines and separation methods are indicated for each panel in the main text figures. Changes in relative abundance were calculated from non-saturated images acquired by scanning phosphor storage screens. Indicated cytosolic rRNAs or tRNAs were detected on the same membranes as mitochondrial RNAs and used as loading controls. RNA variants with short A-tail and long A/U-tail are listed separately.

Supplementary Table S3. Read statistics for eCLAP, global mitochondrial RNA-Seq and targeted Tail-Seq.

Supplementary methods

Parasite maintenance, RNAi, protein expression and RNA analysis

The construct for tetracycline-inducible KPAF5 RNAi knockdown was generated by amplifying a gene fragment with D38/D39 primers and cloning the product into p2T7-177 vector (1). Linearized plasmid was transfected into a procyclic Lister 427 (29-13) and bloodstream (SM) *T. brucei* strains (2). For inducible protein expression, full-length KPAF5 gene were PCR-amplified from genomic DNA using D107/D108 primers and cloned into pLew-79-MHTAP/phleomycin vector (3). Expression was induced with 1 mg/L tetracycline and maintained for 72 hours. For BioID experiments, *MERS1, MERS2, KPAP1, KPAF3, KPAF4, KPAF5, RESC2, RESC5, RESC7, RESC 13, RESC18* and *RESC19* genes were cloned into the pLew-100-V5-Ble vector to generate C-terminal fusions with mutated *E.coli* BirA* ligase. Expression of BirA*-tagged proteins was induced for 48 hours at which point biotin was added to 50 μM, and cultivation continued for 24 hours. DNA oligonucleotides are listed below.

Preparation of mitochondrial lysate, glycerol gradient fractionation and native PAGE

PF parasites expressing TAP-tagged proteins were cultured in 850 ml of SDM-79 media supplemented with 10% FBS, 10 μg/ml hemin, 50 μg/ml G418, 2.5 μg/ml phleomycin and 50 μg/ml Hygromycin B at 27°C. Protein expression was induced for 72 hours with 1 mg/L of tetracycline. Cells were collected by centrifugation at 3000g for 10 min at 4°C, washed in PBS and resuspended in DTE buffer (5 mM Tris-HCl, 1mM EDTA, pH 8.0) at 1.2×10^9 cells/ml. Suspension was passed through 26-gauge needle and quickly mixed with 60% sucrose to achieve 7.2%. Lysate was diluted twice with STE buffer (20 mM Tris-HCl pH 7.6, 250 mM sucrose, 1 mM EDTA), supplemented with 3 mM $MgCl_2$ and 25 U/ml DNAse I (Sigma), and incubated for 20 min on ice. Crude mitochondrial fraction were pelleted by centrifugation at 15,000g for 15 min at 4 °C, washed with 50 ml of STE likewise, and frozen in liquid nitrogen. Mitochondrial pellet was resuspended in lysis buffer (30 mM HEPES, pH 7.3, 120 mM KCl, 12 mM $MgCl_2$, 1 mM DTT, 1/10 of Complete Protease Inhibitor, 2 U of Turbo DNase and 1.2% NP40) at 0.6 g/ml, incubated for 10 min on ice and centrifuged for 15 min at 21,000g and 4°C. The 10%-30% glycerol gradient in 25 mM HEPES pH 7.3, 100 mM KCl and 10 mM $MgCl_2$ was prepared with Gradient Master (Biocomp Instruments). Mitochondrial extract was applied on the gradient surface, centrifuged in SW41 rotor (Beckman) for 5 hours at 141,000g with slow breaking. Eighteen fractions were collected using Gradient Station Fractionator (Biocomp Instruments). Each fraction was mixed with Coomassie R250 to 0.25%, loaded on NativePAGE 3%-12% Bis-Tris Protein Gel (Invitrogen), separated and electroblotted onto nitrocellulose membrane.

Rapid affinity purification

TAP-tagged KPAF1, KPAF2, KPAF3, KPAF4, KPAF5, KPAP1 and MERS2 were purified by rapid protocol from ~1.5 g of crude mitochondria pellet (wet weight). Mitochondrial pellet was pulverized in LN2 with CryoMill (Retsch) and stored at -80°C. Frozen powder was resuspended in 3 ml of pre-warmed lysis buffer (50 mM Tris-HCl, pH7.6, 500 mM NaCl, 12 mM $MgCl_2$, 1% NP-40, 5% glycerol) supplemented with Compete protease inhibitor (Roche), 20 U of Turbo DNase (Ambion) and incubated on ice for 10 min. Extract was diluted 50 mM Tris-HCl, pH7.6, 500 mM NaCl, 12 mM $MgCl_2$, 5% glycerol 4 times and centrifuged at 40000 rpm for 20 min on

ultracentrifuge (Beckman). Cleared extract was filtered through 0.45 μm low protein binding syringe filter, mixed with 10 mg of IgG-coated Dynabeads (ThermoFisher) magnetic beads and incubated on cold with slow rotating for 10 min. Beads were collected on a magnet stand (Life Technologies), washed twice with 6 ml of wash buffer (20 mM Tris-HCl, pH 7.6, 500 mM NaCl, 5 mM EDTA, 0.5% NP-40, 5% glycerol) and 3 times with 10 ml of wash buffer for 5 min on Nutator at 4^0C. Beads were transferred into 1.5 ml low protein binding tube (Eppendorf) and washed twice with 1 ml of wash buffer on cold using Thermo shaker (Eppendorf). Proteins were eluted from beads with 0.3 ml of TEV buffer (20 mM Tris-HCl, pH 8.0, 0.5 mM EDTA, 1 mM DTT) with 20 U of TEV protease and Compete protease inhibitor (Roche) overnight at 4°C.

Purification of recombinant proteins and antibody production

KPAF4 and KPAF5-Strep-Tag were amplified from genomic DNA with D32/D33 and D34/D35 primer pairs and cloned to pETDuet1 vector (Novagen) to generate N-terminally 6-His tagged KPAF4. Transformed BL21 (DE3) STAR *E. coli* cells were grown overnight at 37 ^{0}C in the presence of 100 μg/ml of ampicillin in 2YT media. Overnight culture was diluted 100-fold to 600 mL of 2YT supplemented with 2% glucose and 50 μg/mL of ampicillin and grwon at 37 ^{0}C until 0.8 OD^{600}. Temperature was lowered to 20 ^{0}C for 1 hour and expression was then induced with 1 mM IPTG. Cell were cultivated for 4 hours, harvested by centrifugation at 5000 x g for 10 min, frozen in liquid nitrogen and cryogenically ground by CryoMill (Retsch). Powder was resuspended in 100 ml of lysis buffer (50 mM HEPES (pH 8.0), 300 mM NaCl, 0.1% Triton X-100) supplemented with of 6,000 units/ml of DNase I (Sigma), 0.1 mg/mL of lysozyme (EMD Millipore) and 1 tablet of Complete protease inhibitor cocktail EDTA free (Roche). Extract was sonicated (Virsonic 600) 3 times at 12W for 15 sec, cleared by centrifugation at 30,000 rpm in a SW32Ti rotor for 20 minutes, filtrated through 0.45 μm pore-sized low-protein binding filter and loaded onto 3 ml Talon resin (Clontech) pre-equilibrated in Talon A buffer (25 mM HEPES (pH 7.3) and 300 mM NaCl). The column was washed with 40 mL of Talon A buffer containing 10 mM imidazole. Protein complex was eluted in 10 mL of Talon B buffer (50 mM HEPES (pH 7.3), 300 mM NaCl, and 200 mM imidazole). Eluate was loaded on 1 mL StrepTrap HP column (GE Healthcare) pre-equilibrated with Strep A buffer (50 mM Tris-HCl, pH 8.0,300 mM KCl and 1 mM DTT) at 0.5 ml/min. Column was washed with 20 mL of Strep A buffer and protein complex was eluted in 10 mL of Strep B Buffer (50 mM Tris-HCl, pH 8.0, 1 mM DTT, 2.5 mM desthiobiotin). Peak fractions were concentrated to 100 μl and loaded on Superose 12 GL size exclusion column (GE, Healthcare) equilibrated with 50 mM HEPES, pH 8.0, 200 mM NaCl, 1 mM EDTA and 1 mM DTT.

Individual KPAF5 gene was amplified from genomic DNA using D359/D360 primer pair and inserted into Nco I and Hind III sites of pET28b vector (Novagen) with Gibson Assembly Master Mix (New England Biolab). Cells were grown as described above in 4L of 2YT media. Pellet (~12 g wet weight) was cryogenically ground in CryoMill (Retsch), and resuspended in 200 ml of lysis buffer (50 mM HEPES (pH 8.0), 300 mM NaCl, 0.1% Triton X-100) with of 1,000 units/L of DNase I (Sigma), 0.1 mg/ml of lysozyme (EMD Millipore) and 2 tablet of Complete protease inhibitor cocktail EDTA free (Roche). The lysate was sonicated (Virsonic 600, 3X at 12W for 15 sec), cleared by centrifugation at 30,000 rpm in a SW32Ti rotor for 20 minutes, and filtered with 0.2 μm low-protein binding filter, and loaded onto 3 ml Talon resin (Clontech) column pre-equilibrated with Talon A buffer (25 mM HEPES (pH 7.3) and 300 mM NaCl). The column was washed with 40 ml of Talon A buffer containing 10 mM imidazole after sample loading. Protein was eluted with Talon B buffer (50 mM HEPES (pH 7.3, 300 mM NaCl, and 200 mM imidazole).

The peak fractions were pooled (~16 ml) and 4 ml out of the pooled fractions was diluted 2.5-fold with SP A buffer (25 mM HEPES (pH 7.3, 1 mM EDTA and 1 mM DTT). The diluted proteins were loaded onto 1 ml HiTrap SP column (GE Healthcare) pre-equilibrated with SP A buffer containing 100 mM NaCl and eluted with 20 ml linear gradient of NaCl from 100 to 500 mM. KPAF5 was eluted at ~400 mM of NaCl. The peak fractions containing KPAF5 were pooled, supplemented with glycerol to 10%, and frozen in liquid nitrogen. The purity was over 95 % according to SDS polyacrylamide gel analysis. Purified KPAF5 was used for rabbit immunization and serum collection at Covance Inc. For antibody affinity purification, the crude IgG ffraction was isolated from rabbit antiserum and passed through antigen column coupled with KPAF5 as follows. Purified KPAF5 was dialyzed against PBS and then against coupling buffer containing 0.2 M NaHCO3 and 0.5 M NaCl. The protein (~3 mg) was concentrated to 700 µl and loaded into to 1ml of HiTrap NHS-activated HP column (GE Healthcare) and incubated for 30 min at room temperature. The ligand-coupled resin was transferred from the column into a 15 ml conical tube with 5 ml of buffer A (0.5 M ethanolamine and 0.5 M NaCl (pH 8.0)) and then transferred into 1 ml disposable polystyrene column (Pierce). The transferred resin was washed with 6 ml of buffer B (0.1 M sodium acetate and 0.5 M NaCl (pH 4)), incubated with 6 ml of buffer A, washed with 6 ml of buffer B, 6 ml of buffer A, 6 ml of buffer B, and 6 ml of 1xTBS. To prepare crude IgG sample, 7.5 ml of saturated ammonium sulfate was added to 15 ml of the antiserum in 1 ml increments with swirling, and incubated on ice for 30 min. The mixture was centrifuged at 15,000 x g for 15 min at 4 °C. The supernatant was recovered into a new 50 ml conical tube, supplemented with 9 ml of saturated ammonium sulfate with swirling, and incubated on ice for 30 min. The mixture was centrifuged at 15,000 x g for 15 min at 4 oC, and the pellet was resuspended in 1 ml of 10 mM Tris-HCl (pH 8) and desalted with PD-10 column (GE Healthcare) pre-equilibrated with 1xTBS. The eluate was transferred into the prepared antigen column and mixed on Nutator mixer for 1 hour at room temperature. The column was washed with 50 ml of antibody wash buffer (20 mM Tris-HCl (pH 7.6, 0.5 M NaCl). The bound IgG was eluted with 1.2 ml of 100 mM glycine (pH 2.5) and immediately mixed with 0.3 ml of 1 M Tris-HCl (pH 8). Six fractions (9 ml) were collected and dialyzed against 300 ml of storage buffer containing 1xPBS, 1 mM EDTA and 50% glycerol at 4 °C for 16 hours and stored at -20 °C.

Coupled *in vitro* transcription-translation in reticulocyte lysate and immunoprecipitation

KPAF5 and KPAF4 proteins were co-synthesized *in vitro* from individual T7 promoter-containing plasmid following TNT protocol (Promega) using 100 ng of plasmid and 5 µCi of [^{35}S] methionine. Products were separated on 8-16% Tris-Glycine gel (Novex) in SDS running buffer, transferred on nitrocellulose membrane, expose to phosphor screen and visualized by scanning on Phosphorimager (FLA7000 Typhoon, GE). Co-precipitation were perform using 0.45 µg Dynabeads Protein G (Thermo Fisher) conjugated with 2 µg of KPAF5 polyclonal antibody by following manufacture protocol. TNT reactions were incubated with magnetic beads at 15^{0}C for 30 min, beads were collected on magnetic stand and washed with 20 mM Tris-Hcl pH7.5, 150 mM KCl, 0.2% Tween, 0.5 mg/ml BSA, 5% Glycerol Buffer. Proteins bound to beads were eluted with SDS sample buffer, separated on 8-16% Tris-Glycine gel in SDS running buffer and transferred on nitrocellulose membrane. Protein bands were visualized as described previously.

RNA isolation

PF and BF cell cultures (~25 ×10^7 cells) were centrifuged at 3000 g for 10 min at 4°C, cells washed with 50 ml of cold PBS, transferred to 2 ml tube, and re-pelleted at 3000 g for 5 min at 4°C. Cell

pellets were resuspended in 0.8 ml of cold Solution D (4M guanidine isothiocyanate, 25 mM sodium citrate, pH 7.0, 0.5% sarcosyl, 0.1 M 2-mercaptoethanol), supplemented with 0.1 ml of 2 M sodium acetate (pH 4.0), gently diluted with 0.9 ml of water-saturated phenol pH 6.6 and mixed with 0.3 ml of Chloroform. Phases were separated by centrifugation at 21,000 x g for 10 min at 4 ^{0}C. Upper phase was transferred in a Phase Lock Gel Heavy 2-ml tube (5Prime) and RNA extracted vortexing for 1 min with 0.8 ml of chloroform. Phases were separated by centrifugation for 5 min at 21,000.0g and room T ^{0}C. RNA was precipitated with 1 ml of isopropanol and re-precipitated with ethanol. RNA was treated with DNase I (Invitrogen) for northern blotting and circularization and Turbo DNase (Ambion) for Real Time PCR.

Quantitative RT-PCR

cDNA was synthesized from 2 µg of TURBO DNase-treated and column (RNA Cleanup, Qiagen) purified total RNA with TagMan Reverse Transcription Reagents (N808-0234, Applied Biosystems) following company recommendation. PCR reaction was set up by mixing Power SYBR Green Master Mix with primers at 1.5 µM concentration and cDNA. Triplicate aliquots were distributed into 96 well plate (951022043, Eppendorf). PCR was performed in Eppendorf Realplex 2S cycler as follows: 95°C, 10 min; 95°C (15 s), 60°C (1 min, measure point), 45 cycles.

RNA circularization and reverse transcription

Total RNA (~5 µg, qRT-PCR grade) was mixed in 50 µl reaction with 30 U of T4 RNA ligase 1 (New England Biolabs), 1 mM ATP, 5 mM DTT, 40 U of RNase OUT (Thermo Fisher Scientific), 10% of PEG 8000 and incubated at 14°C overnight, followed by incubation for 30 min at 37°C. Remaining linear RNA was digested with 0.1 U/µl of RNase R (Epicenter) for 10 min at 37°C. Circular RNA was extracted by phenol/chloroform and precipitated with ethanol. cDNA was synthesized using 2.5 µg of circular RNA in 20 µl reaction containing 20 U/µl of SuperScript III Reverse Transcriptase (Thermo Fisher Scientific) and 1µM gene-specific primer (Table below) following company recommendation. RNA was digested with 1 U of RNase H (Thermo Fisher Scientific), 1 µl of RNace-IT RNase cocktail (Agilent) for 10 min at 37°C and column purified.

RNA-Seq library preparation

The libraries were generated in two sequential PCR reactions. First PCR was performed in a 100 µl reaction in the presence of 500 pmol of each transcript-specific primer using 5 µl of the cDNA synthesis reaction and 2 U of Phusion Hot Start II DNA Polymerase (Thermo Fisher Scientific) according to manufacturer recommendations. After an initial denaturation step of 30 s at 98°C, the PCR reactions underwent five three-step PCR cycles (98°C 10 s, 55°C 30 s, 72°C 20 s) and five additional two-step PCR cycles with higher annealing temperature (98°C 10 s, 72°C 30 s) followed by final elongation step of 1 min at 72°C. PCR products were purified with DNA Clean and Concentrator-5 kit (Zymo Research) and eluted in 20 µl of 10 mM Tris-HCl, pH 8.0. Second PCR was performed in 100 µl reaction with the presence of 600 pmol of each Illumina universal forward primer and Illumina indexed reverse primers, using 10 µl of the purified first PCR reaction and Phusion Hot Start II DNA Polymerase (Thermo Fisher Scientific). After an initial denaturation step of 30 s at 98°C, the PCR reactions underwent 16 PCR cycles (98°C 10 s, 60°C 30 s, 72°C 20 s) and a final elongation step of 1 min at 72°C. After purification of the PCR products on a ZYMO DNA clean and concentrator-5 column and elution in 12 µl, PCR products were resolved on a 6% acrylamide/TBE gel. After staining the gel with SYBR Green 1, the area of interest was excised under blue light. PCR products were extracted with 5 volumes of extraction buffer (0.5 M

ammonium acetate, 0.1% SDS and 1 mM EDTA) for overnight at room temperature on a Nutator. The supernatant was concentrated 5 - 6 times with 2-butanol, DNA was extracted with phenol/chloroform, precipitated with ethanol, and cleaned-up using DNA Clean and Concentrator-5 kit (Zymo Research). Size distribution of the libraries was analyzed by 2100 Bioanalyzer (Agilent). Deep sequencing of the libraries was carried out on Illumina platform.

eCLAP (UV-crosslinking, two-steps affinity purification, library preparation and sequencing)

T. brucei cells were seeded at 10^6 cells/ml in 850 ml of SDM-79 medium supplemented with 10% FBS. Expression of TAP-tagged protein was induced with 1 μg/ml of tetracycline, and the cells were grown to ~20 x10^6 cells/ml for 72 hours post-induction. Cell culture was exposed to 254 nm UV light for 20 seconds in Vari-X-link instrument. Irradiated culture was collected by centrifugation at 3000g for for10 min and washed with PBS. Cell pellet was resuspended in 3 ml of Extraction Buffer (50 mM Tris-HCl, pH 7.6. 150 mM NaCl, 2 mM MCl_2, 1%NP40, 1/20 of Complete Protease Inhibitor tablet without EDTA, 40U of TURBO DNase) and incubated on ice for 10 min. Extract was diluted with 50 mM Tris-HCl, pH 7.6. 150 mM NaCl, 2 mM MCl_2 buffer to 11 ml, sonicated 3 times for 20 s at 12W. Extract was centrifuged at 40,000 rpm for 20 min using SW41 rotor. The supernatant was filtered through 0.22 μn low protein binding filter. RNA was partially digested by incubation with 40 U/ml of RNase I for 5 min at 37°C in the presence of 5 mM EDTA. Sample was mixed with IgG sepharose (GE, Life Sciences), incubated for 30 min at 4 ^{0}C on nutator. Bound proteins were washed three times with IgG-wash buffer (20 mM Tris-HCl pH 7.6, 150 mM NaCl, 0.1%NP40), 2 times with IgG-wash buffer containing 500 mM NaCl and eluted with Ni-Binding Buffer (6M Guanidium Chloride, PBS pH7.6, 10 mM Imidazole). Eluate was mixed with 0.5 mg of His-Pur Ni-NTA magnetic beads (Thermo Fisher Scientific) and incubated at 25 ^{0}C and 100 RPM shaking (Eppendorf Thermo shaker) overnight. All manipulations with beads were performed on magnetic stand. Beads were washed three times with Ni-Binding Buffer for 5 min at 1000 rpm at 25°C, three times with TAP buffer (10 mM Tris-HCl pH 7.5, 5 mM $MgCl_2$, 100 mM KCl, 0.02% Triton-X100) at 10 ^{0}C and incubated at 37 ^{0}C with 8U of TAP (Thermosensitive Alkaline Phosphatase) in TAP buffer containing 5U of DNase (New England Biolabs) and 1 U of Murine RNase inhibitor. Ligation of the 4 μM InvRil19 RNA 3′ adaptor (5-/5Phos/rArGrA rUrCrG rGrArA rGrArG rCrArC rArCrG rUrC/3SpC3/-3′) was performed by incubation beads at 25 ^{0}C and 1200 RPM for 75 min in the buffer containing 10 mM ATP, 3% DMSO, 15% PEG 8000, 1 U Marine RNase, 100U of Hi-T4 ligase (New England Biolabs). Beads were washed three times with PNK buffer (40 mM Tris-HCl, pH 7.5, 10 mM $MgCl_2$, 0.1% NP40) and incubated in PNK buffer containing 20U of T4 Polynucleotide kinase (Thermo Fisher Scientific), 1 μCi of γ-ATP (Perkin Elmer), 10 U of Murine RNase inhibitor (New England Biolabs) for 10 min at 37 ^{0}C at 800 RPM. Bound RNA-protein complex was washed three times with PNK buffer, eluted with LDS-MOPS loading buffer and 200 mM Imidazole, separated on 4-12% NuPAGE gel (Thermo Fisher Scientific) and transferred on nitrocellulose membrane. Crosslinked complexes were visualized on Phosphorimager (FLA7000 Typhoon, GE) as described before. Radioactive band were cut form membrane and used to extract RNA. RNA was isolated by incubation of membrane pieces at 37 ^{0}C for 30 min in PK buffer (100 mM Tris-HCl, pH 7.5, 50 mM NaCl, 10 mM EDTA) containing 4 mg/ml of protease K, water- saturated phenol and following chloroform extractions, ethanol precipitation with 5 μg glycogen and purification using RNA cleanup kit (Zymo). cDNA was synthetized using 10 μM of InvAR17 primer (5'-CAG ACG TGT GCT CTT CCG A-3') and 100 U TGIRT Reverse Transcriptase (InGen) by incubation at 55

^{0}C for 30 min followed by 60 ^{0}C for 30 min in the buffer containing 5 mM DTT, 50 mM NaCl, 3 mM $MgCl_2$, 20 mM Tris-HCl pH 7.5, 0.5 mM dNTP mix and 10U RNase out. cDNA was subjected to ExoSAP-IT (Thermo Fisher Scientific) treatment for 15 min at 37 ^{0}C, NaOH hydrolysis at 70 ^{0}C for 12 min and following neutralization with HCl. cDNA was purified using MyOne Silane beads (Thermo Fisher Scientific). Ligation of 4 µM of InvRandd3T3 5' adaptor (5'-/5Phos/NNN NNN NNN NAG ATC GGA AGA GCG TCG TGT /3SpC3/-3') was performed at 22 ^{0}C overnight using the buffer for 5′ adaptor ligation described before. cDNA was purified with MyOne Silane beads and used in HotStart polymerase (Thermo Fisher Scientific) to sreat library for HiSeq sequencing (GENEWIZ).

Oligonucleotides table. P, pre-edited; E, edited; FW, forward; RV, reverse.

Cloning Primers	sequence 5’ -3’
D39 RNAi	ATAGGATCCGGGGCCGCGGGGTCTCGCGA
D38 RNAi	GAGAAGCTTGTGTGCGTGTGTGCATTCTG
D107 MHTAP	CAGAAGCTTATGCAGAGACTCTTTTTAGATCGACATG
D108 MHTAP	TAAGGATCCTTATTTCTGCACTGTGGGCGTCC
C997 RESC2-BirAstar	ACCAAAAAGTAAAATTCACAATGCTGCGCGCGCGCCTG
C998 RESC2-BirAstar	TGAGTTTTTGTTCCATATGAAGCTTGTATGCCGAAACGGCAGTCATG GTGTG
D143 RESC5-BirAstar	TGAGTTTTTGTTCCATATGAAGCTTTTTCTGCAGTTGATGCGTCTG
D142 RESC5-BirAstar	ACCAAAAAGTAAAATTCACAATGCTGCGCCACACATCAC
D145 RESC7-BirAstar	TGAGTTTTTGTTCCATATGAAGCTTCTGTGTACCGTGTTCACCC
D144 RESC7-BirAstar	ACCAAAAAGTAAAATTCACAATGAGGAGTAGCCGGGGTATTTTG
D127 RESC13-BirAstar	TGAGTTTTTGTTCCATATGAAGCTTCACCTTCTGACTGGCATC
D119 RESC13-BirAstar	ACCAAAAAGTAAAATTCACAATGAAGCGCACACCTGTTAG
D123 RESC18-BirAstar	ACCAAAAAGTAAAATTCACAATGCGGACGTTCCTGGCA
D124 RESC18-BirAstar	TGAGTTTTTGTTCCATATGACAGCGGAACAAACTGTTTCTGAG
D9 MERS1-BirAstar	TGAGTTTTTGTTCCATATGAAGCTTCGATGCATCTTCCCCGTTAC
D8 MERS1-BirAstar	ACCAAAAAGTAAAATTCACAATGCGCAAGCAATTATTTTTC
D10 MERS2-BirAstar	ACCAAAAAGTAAAATTCACAATGACATCTAGCGTAACCG
D11 MERS2-BirAstar	TGAGTTTTTGTTCCATATGAAGCTTTTTTTTCTTCGCCTTGTTC
D14 MERS3-BirAstar	ACCAAAAAGTAAAATTCACAATGAAGAAAGTATGGGCTCAAC
D15 MERS3-BirAstar	TGAGTTTTTGTTCCATATGAAGCTTGCCTTCGCACCAAACTAAG
D6 KPAP1-BirAstar	ACCAAAAAGTAAAATTCACAATGAGAAAGTTTTCAGCTTTTC
D7 KPAP1-BirAstar	TGAGTTTTTGTTCCATATGAAGCTTCTGATGATTGTCGCATTTTATC
D1 KPAF1-BirAstar	TGAGTTTTTGTTCCATATGAAGCTTAACATCCGCACCGGCAGC
C999 KPAF1-BirAstar	ACCAAAAAGTAAAATTCACAATGTTCCAACGCCGGTTAGTG
D2 KPAF3-BirAstar	ACCAAAAAGTAAAATTCACAATGAAACTACTGCGACGAG
D2 KPAF3-BirAstar	TGAGTTTTTGTTCCATATGAAGCTTATGTGCACGATCATCTGC
D4 KPAF4-BirAsrar	ACCAAAAAGTAAAATTCACAATGCTTTCACAGGCTCGC
D5 KPAF4-BirAstar	TGAGTTTTTGTTCCATATGAAGCTTCCCTTTCCCTTCCTCCTC
D84 KPAF5-BirAstar	ACCAAAAAGTAAAATTCACAATGCAGAGACTCTTTTTAGATCGAC
D85 KPAF5-BirAstar	TGAGTTTTTGTTCCATATGAAGCTTTTTCTGCACTGTGGGCGTC
D123 RESC18-BirAstar	ACCAAAAAGTAAAATTCACAATGCGGACGTTCCTGGCA
D124 RESC18-BirAstar	TGAGTTTTTGTTCCATATGACAGCGGAACAAACTGTTTCTGAG
D89 KPAF5 clone to pET28b	GAAGGATCCTTATTTCTGCACTGTGGGCGTCC
D88 KPAF5 clone to pET28b	CAGCATATGCAGAGACTCTTTTTAGATCGACATG
D361 KPAF4-strep-pET28b	CTTTAAGAAGGAGATATACATGGCAATGCTTTCACAGG
D362 KPAF4-strep-pET28b	TCGAGTGCGGCCGCATCATTTTTCAAACTGCGGATGGCTCCAGCCGG ACGATCCTGCCGCCCCTTTCCCTTCCTCCTC
D32 KPAF4 to clone in pET-Duet1/His-Tag	ATAGGATCCGATGGCAATGCTTTCACAGGCTC

D33 KPAF4 to clone in pET-Duet1/His-Tag	GAGAAGCTTTTACCCTTTCCCTTCCTCCTCC
D35 KPAF5 to clone in pET-Duet1/Strep-Tag	CAGCCTAGGTCATTTTTCAAACTGCGGATGGCTCCACTCGAGTTTCT GCACTGTGGGCGTCCCGTCTTCGG
D35 KPAF5 to clone in pET-Duet1/Strep-Tag	CAGCATATGCAGAGACTCTTTTTAGATCGACATG
**Nothern probe primers**	**sequence 5' -3'**
PCR probe RPS12 pre	CGACGGAGAGCTTCTTTTGAATA
PCR probe RPS12 pre	CCCCCCACCCAAATCTTT
PCR probe RPS12 ed	CGTATGTGATTTTTGTATGGTTGTTG
PCR probe RPS12 ed	ACACGTCGGTTACCGGAACT
PCR probe A6 pre	TTGCCTTTGCCAAACTTTTAGAAG
PCR probe A6 pre	ATTCTATAACTCCAAAATCACAACTTTCC
PCR probe A6 ed	CAAACCAACAAACAAATACAAATCAAAC
PCR probe A6 ed	GATTTATTTTGGTTGCGTTTGTTATTATG
A397 Oligo probe CYB pre	GACCCTTTCTTTTTTCTCCGC
B372 Oligo probe CYB ed	TGACATTAAAAGACAACACAAATTTCTAAATAATAAAAAAAATAAT AAAAATCTACAACGAAACATATTTATATAAAATTTATAACC
Oligo probe 9S rRNA	ACGGCTGGCATCCATTTC
Oligo probe 12S rRNA	TGAACAATCAATCATGGTAATAAGTAGACGATG
PCR probe CO1 forward	TGCCTATAACTATGGGTGGGTTTACAAAC
PCR probe CO1 reverse	ACTAAGCAACCAAATCCTCCAATAAACATTC
PCR probe ND1 forward	GGACTGCTTCTTGATGGATTACGTTTACC
PCR probe ND1 reverse	AGATAATTCAGTAACAAGGCCAGCAACAAG
PCR probe Murf5 forward	TGTTCCTTATTCATTTTGTGCATTAC
PCR probe Murf5 reverse	TGTGTATAATGTTAAGTCAAATTAAAATGC
A504 9S oligo probe	ACGGCTGGCATCCATTTC
A304 12S oligo probe	TGAACAATCAATCATGGTAATAAGTAGACGATG
A851 5.8S oligo probe	GGAAGCCAAGTCATCCATCGCGACACGTTGTGGGAGCCGTGG
A343 18S oligo probe	TGGTAAAGTTCCCCGTGTTGA
B108 A6(14) gRNA oligo probe	ATAATTATCATATCACTGTCAAAATCTGATTCGTTATCGGAGTTATA GTATAT
B110 Cyb(560) gRNA oligo probe	GTAGTTATCCTCCCCATTACTCAGAAATCTACATTGTCTTTTACTATC TC
A662 Murf2(II) gRNA oligo probe	CATTCAATTACTCTAATTTAATTTTATTTTTGTGC
A872 $tRNA^{Cys}$ oligo probe	GGGGACCATTCGGACTGCAGCCG
C837 KPAF4 riboprobe	CGATGTTAATACGACTCACTATAGGGCAGCGGTGTATGTGAAG
A883 KPAF4 riboprobe	ATATGAGGTTGGCAGATGTAC
D55 qPCR for KPAF5	CGGCCACGAGAGGTTCCCAT
D54 qPCR for KPAF5	CTGGCCGTGCTTGTGGATGC
**Real Time PCR primers**	**sequence 5' -3'**
A504 qPCR for 9S	ACGGCTGGCATCCATTTC
A503 qPCR for 9S	ATTAGATTGTTTTGTTAATGCTATTAGATG
A304 qPCR for 12S	TGAACAATCAATCATGGTAATAAGTAGACGATG
A303 qPCR for 12S	GGGCAAGTCCTACTCTCCTTTACAAAG
A296 qPCR for ND1	AGATAATTCAGTAACAAGGCCAGCAACAAG
A295 qPCR for ND1	GGACTGCTTCTTGATGGATTACGTTTACC
A298 qPCR for ND4	TGCTATAAATACTAAACCCAACACAATTACACTATC
A297 qPCR for ND4	CAATCTGACCATTCCATGTGTGACTACC
A299 qPCR for ND5	TTTCTATATGTTTGTTAGTAGGATGTGCGTTC
A300 qPCR for ND5	GCGTGTATTAATGCTGATACTGGGATAGG
A294 qPCR for Murf1	AAAGCCAATACAAATACAAAGGTAACTTAG
A293 qPCR for Murf1	GTTTACTACTTGCATGTCTCTTTCTTTG

A526 qPCR for Murf5	TGTGTATAATGTTAAGTCAAATTAAAATGC
A525 qPCR for Murf5	TGTTCCTTATTCATTTTGTGCATTAC
A302 qPCR for CO1	ACTAAGCAACCAAATCCTCCAATAAACATTC
A301 qPCR for CO1	TGCCTATAACTATGGGTGGGTTTACAAAC
A313 qPCR for CO2 pre	TTCATTACACCTACCAGGTTCTCT
A313 qPCR for CO2 pre/ed	ATTACAGTGTAACCATGTATTGACATT
A314 qPCR for CO2 edited	ATTTCATTACACCTACCAGGTATACAA
A346 qPCR for Murf2 pre	GATTTTAAGATTGGCTTTGATTGA
A347 qPCR for Murf2 pre/ed	AATATAAAATCTAGATCAAACCATCACA
A348 qPCR for Murf2 pre	GATTTTAATGTTTGGTTGTTTTAATTTAG
A208 qPCR for Cyb pre/ed	CCCATATATTCTATATAAACAACCTGACA
A209 qPCR for Cyb edited	AAATATGTTTCGTTGTAGATTTTTATTATTT
A207 qPCR for Cyb pre	ATATAAAAGCGGAGAAAAAAGAAAG
A213 A6 edited	CAAACCAACAAACAAATACAAATCAAAC
A213 A6 edited	GATTTATTTTGGTTGCGTTTGTTATTATG
A211 A6 pre-edited	ATTCTATAACTCCAAAATCACAACTTTCC
A210 A6 pre-edited	TTGCCTTTGCCAAACTTTTAGAAG
A358 RPS12 edited	ACACGTCGGTTACCGGAACT
A357 RPS12 edited	CGTATGTGATTTTTGTATGGTTGTTG
A360 RPS12 pre-edited	CCCCCCACCCAAATCTTT
A359 RPS12 pre-edited	CGACGGAGAGCTTCTTTTGAATA
B293 ND3 edited	ACAAATAATGGAATTTAACAATACA
B292 ND3 edited	CGTTGTTGTTTGTGGTTT
A352 ND3 pre-edited	AACAAATCTCTTTACCCCCTTCAG
A351 ND3 pre-edited	GAATGGGAGATGGGTTTTGG
A354 ND7 edited	CTGTACCACGATGCAAATAACCTATAAT
A353 ND7 edited	GCATCCCGCAGCACATG
A356 ND7 pre-edited	GATCTACGGTCCCCTCTTTCCT
A357 ND7 pre-edited	GCGGGCGGAGCATTATT
B266 ND8 edited	GTTGTATTGCTTGTCGTT
B267 ND8 edited	ACAATGAATGCGTAATGG
B183 ND8 pre-edited	AAGCCCATTTTGAGCAGGAG
B184 ND8 pre-edited	TTGGCAAAAATCTGTCGGGC
B190 ND9 edited	AACATTCGCGTTCCAACAAC
B189 ND9 edited	TTGTTTTTGTGTGTTCGTTTGTG
B198 ND9 pre-edited	TTAAGGTTGCCCTGTTGTCG
B197 ND9 pre-edited	AACATCGAGGAGTTTTGGGG
A205 CO3 edited	TTGTGTTTTATTACGTTGTATCCAGTATTG
A206 CO3 edited	CGAAAGCAAACTCACAACACAAA
A288 CO3 pre-edited	GGGAAACCAGATGAGATTG
A289 CO3 pre-edited	ACTACCTCTTCATTCCAACTA
A343 18S	TGGTAAAGTTCCCCGTGTTGA
A342 18S	CGGAATGGCACCACAAGAC
A344 β-tubulin	TTCCGCACCCTGAAACTGA
A345 β-tubulin	TGACGCCGGACACAACAG
**Tail sequencing primers**	**sequence 5' -3'**
A478 RPS12 pre RT	GGCTGGGCTCGAACGGCTC
D323 RPS12 pre hiSeq PCR	GTTCAGAGTTCTACAGTCCGACGATCCCTTTGTTTTGGTTAAAGAAA CATCGTTTAGAAGA
D324 RPS12 pre hiSeq PCR	AGACGTGTGCTCTTCCGATCTTCTCCAGTCCCACTCAAAAAATCCTC
C578 A6 pre RT	CAAAACCTTTCTCCTTCATTTCCTCTCC
D325 A6 pre hiSeq PCR	GTTCAGAGTTCTACAGTCCGACGATCAGGAGAGAAGAAAGGGAAAG TTGTGA
D326 A6 pre hiSeq PCR	AGACGTGTGCTCTTCCGATCTTCCTTCATTTCCTCTCCTGTCTCC

D327 Cyb pre RT	TACCCCATATATTCTATATAAACAACCTG
D328 Cyb pre hiSeq PCR	GTTCAGAGTTCTACAGTCCGACGATCAGTATTATTATTATTTTTGTTG ATAGTGTGTAGG
D329 Cyb pre hiSeq PCR	AGACGTGTGCTCTTCCGATCTAAACAACCTGACATTAAAAGACCCT
C865 CO1 RT	ATAAATAAGGAGTAAATATAACCGATAAATCC
C945 CO1 hiSeq PCR	GTTCAGAGTTCTACAGTCCGACGATCATATTTTTTTTGACAAGCCTT CC
D330 CO1 hiSeq PCR	AGACGTGTGCTCTTCCGATCTTAACCGATAAATCCACATAAAATAGC
A863 9S RT	CAAAATTATAAATTATTATTAACTATTCC
D331 9S hiSeq PCR	GTTCAGAGTTCTACAGTCCGACGATCACCAATTTTTATAATAAAAAT AACGTGCAG
D332 9S hiSeq PCR	AGACGTGTGCTCTTCCGATCTTATACAAAAAATCTTTCAAAAATAAA CC
A866 12S RT	CTATAAATATGTATCTAAATATAAAACAAC
D333 12S hiSeq PCR	GTTCAGAGTTCTACAGTCCGACGATCTTATAATTAATAATAATTAAT ATAAGTACGCAAGG
D334 12S hiSeq PCR	AGACGTGTGCTCTTCCGATCTAAATAATACAAATAATAAATTTACTA CACGG

References

1. Wickstead, B., Ersfeld, K. and Gull, K. (2002) Targeting of a tetracycline-inducible expression system to the transcriptionally silent minichromosomes of Trypanosoma brucei. *Mol. Biochem. Parasitol*, **125**, 211-216.
2. Wirtz, E., Leal, S., Ochatt, C. and Cross, G.A. (1999) A tightly regulated inducible expression system for conditional gene knock-outs and dominant-negative genetics in Trypanosoma brucei. *Mol. Biochem. Parasitol*, **99**, 89-101.
3. Jensen, B.C., Sivam, D., Kifer, C.T., Myler, P.J. and Parsons, M. (2009) Widespread variation in transcript abundance within and across developmental stages of Trypanosoma brucei. *BMC Genomics*, **10**, 482.
